# The PSMA8 subunit of the spermatoproteasome is essential for proper meiotic exit and mouse fertility

**DOI:** 10.1371/journal.pgen.1008316

**Published:** 2019-08-22

**Authors:** Laura Gómez-H, Natalia Felipe-Medina, Yazmine B. Condezo, Rodrigo Garcia-Valiente, Isabel Ramos, José Angel Suja, José Luis Barbero, Ignasi Roig, Manuel Sánchez-Martín, Dirk G. de Rooij, Elena Llano, Alberto M. Pendas

**Affiliations:** 1 Molecular Mechanisms Program, Centro de Investigación del Cáncer and Instituto de Biología Molecular y Celular del Cáncer (CSIC-Universidad de Salamanca), Salamanca, Spain; 2 Unidad de Biología Celular, Universidad Autónoma de Madrid, Madrid, Spain; 3 Centro de Investigaciones Biológicas (CSIC), Madrid, Spain; 4 Genome Integrity and Instability Group, Institut de Biotecnologia i Biomedicina, Universitat Autònoma de Barcelona, Cerdanyola del Vallès, Spain; 5 Departamento de Medicina, Universidad de Salamanca, Salamanca, Spain; 6 Reproductive Biology Group, Division of Developmental Biology, Department of Biology, Faculty of Science, Utrecht University, Utrecht, The Netherlands; 7 Center for Reproductive Medicine, Academic Medical Center, University of Amsterdam, Amsterdam, The Netherlands; 8 Departamento de Fisiología y Farmacología, Universidad de Salamanca, Salamanca, Spain; The Jackson Laboratory, USA, UNITED STATES

## Abstract

The ubiquitin proteasome system regulates meiotic recombination in yeast through its association with the synaptonemal complex, a ‘zipper’-like structure that holds homologous chromosome pairs in synapsis during meiotic prophase I. In mammals, the proteasome activator subunit PA200 targets acetylated histones for degradation during somatic DNA double strand break repair and during histone replacement during spermiogenesis. We investigated the role of the testis-specific proteasomal subunit α4s (PSMA8) during spermatogenesis, and found that PSMA8 was localized to and dependent on the central region of the synaptonemal complex. Accordingly, synapsis-deficient mice show delocalization of PSMA8. Moreover, though *Psma8*-deficient mice are proficient in meiotic homologous recombination, there are alterations in the proteostasis of several key meiotic players that, in addition to the known substrate acetylated histones, have been shown by a proteomic approach to interact with PSMA8, such as SYCP3, SYCP1, CDK1 and TRIP13. These alterations lead to an accumulation of spermatocytes in metaphase I and II which either enter massively into apoptosis or give rise to a low number of aberrant round spermatids that apoptose before histone replacement takes place.

## Author summary

Proteins within the cells that are unnecessary or damaged are degraded by a large protein complex named the proteasome. The proteins to be degraded are marked by a small protein called ubiquitin. The addition of a small modification (acetyl group) to some proteins also promotes their degradation by the proteasome. Proteasomal degradation of proteins is an essential mechanism for many developmental programs including gametogenesis, a process whereby a diploid cell produces a haploid cell or gamete (sperm or egg). The mechanism by which this genome reduction occurs is called meiosis. Here, we report the study of a protein, named PSMA8 that is specific for the testis proteasome in vertebrates. Using the mouse as a model, we show that loss of PSMA8 leads to infertility in males. By co-immunoprecipitation-coupled mass spectroscopy we identified a large list of novel PSMA8 interacting proteins. We focused our functional analysis on several key meiotic proteins which were accumulated such as SYCP3, SYCP1, CDK1 and TRIP13 in addition to the known substrate of the spermatoproteasome, the acetylated histones. We suggest that the altered accumulation of these important proteins causes a disequilibrium of the meiotic division that produces apoptotic spermatocytes in metaphase I and II and also early spermatids that die soon after reaching this stage.

## Introduction

Intracellular protein content is controlled through the balance between the rates of their synthesis and degradation. In eukaryotic cells, the bulk of the degradation is carried out by the ubiquitin-proteasome system (UPS). The proteasome is a multi-subunit complex that eliminates proteins, typically labeled with ubiquitin, by ATP-driven proteolysis [1]. Proteasome complexes comprise a cylindrical catalytic core particle (CP, 20S) and different regulatory particles (RPs, 19S) that regulate the access to the CP by capping it at either end [2]. The CP is composed of seven α-type subunits and seven β-type subunits arranged as a cylinder of four rings (α1–7, β1–7, β1–7, α1–7) [1, 3]. RPs are composed of 20 subunits and their association with the CP is ATP-dependent. There are four additional activators, the 11S regulator PA28α/β/γ and the ubiquitous PA200 (*Psme4*) regulator that stimulates protein degradation independently of ubiquitin [4] and plays a main role in acetylation-dependent degradation of somatic core histones during DNA repair and spermiogenesis [5, 6]. Hybrid proteasomes enclosing a RP at one end and an activator at the other end are also possible [7]. In addition, there are paralogs for three β-genes that are expressed only in the immunological system, which constitutes the immunoproteasome [8], and one β5t gene expressed exclusively in the thymus, which constitutes the thymoproteasome [9]. Finally, there is a meiotic paralog of the α4 subunit (*Psma7*), named α4s (*Psma8)* [10], which might provide substrate specificity and heterogeneity to the α4s-cotaning proteasome.

The proteolytic activity of the proteasome is regulated by the rate of protein ubiquitylation, but also by its association with E3 ubiquitin ligases and deubiquitinating enzymes that edit their potential substrates [11, 12]. The classical targets of the UPS are misfolded or damaged proteins and/or short-lived regulatory proteins, whose concentration is regulated by fine-tuning of their synthesis and degradation kinetics [13, 14]. Typical examples of the latter proteins are cyclins [15, 16]. More recently, it has been hypothesized but not proven that the ZMM complex (also known as the synapsis initiation complex) involved in meiotic homologous recombination is similarly regulated in the mouse [17, 18].

Meiosis is a fundamental process in sexually reproducing species that ensures the production of genetic diversity and the generation of haploid gametes from diploid progenitors [19]. This reduction in genome content is achieved by the physical connections between homologs by chiasmata [20], which are mediated by the repair of self-induced DNA double-strand breaks (DSBs) as crossing-overs (COs). Meiotic recombination takes place on proteinaceous core structures or axial elements (AEs) that scaffold the chromosomal DNA content and physically connect (synapse) homologs through the assembly of the synaptonemal complex (SC) during prophase I [21].

The UPS regulates meiotic recombination in yeast and mouse *via* its physical association to AEs [17, 22]. Given the unknown function that the α4s-containing proteasome plays during spermatogenesis, we explored its function in the mouse. In this study, we show that PSMA8 is localized to and dependent on the central element of the SC, and promotes the assembly of the proteasome activator PA200. Accordingly, synapsis-deficient mice show delocalization of PSMA8. Also, *Psma8*-deficient mice are proficient in meiotic homologous recombination, but show alterations in the proteostasis of several key meiotic players including acetylated histones, SYCP3, SYCP1, CDK1 and TRIP13, which in turn leads to an aberrant meiotic exit, accumulation of apoptotic spermatocytes in metaphase I and II, and finally early spermatid arrest long before histone replacement takes place.

## Results

### PSMA8 is expressed in spermatocytes and its localization to the SC is dependent on synapsis

**Fig 1:**
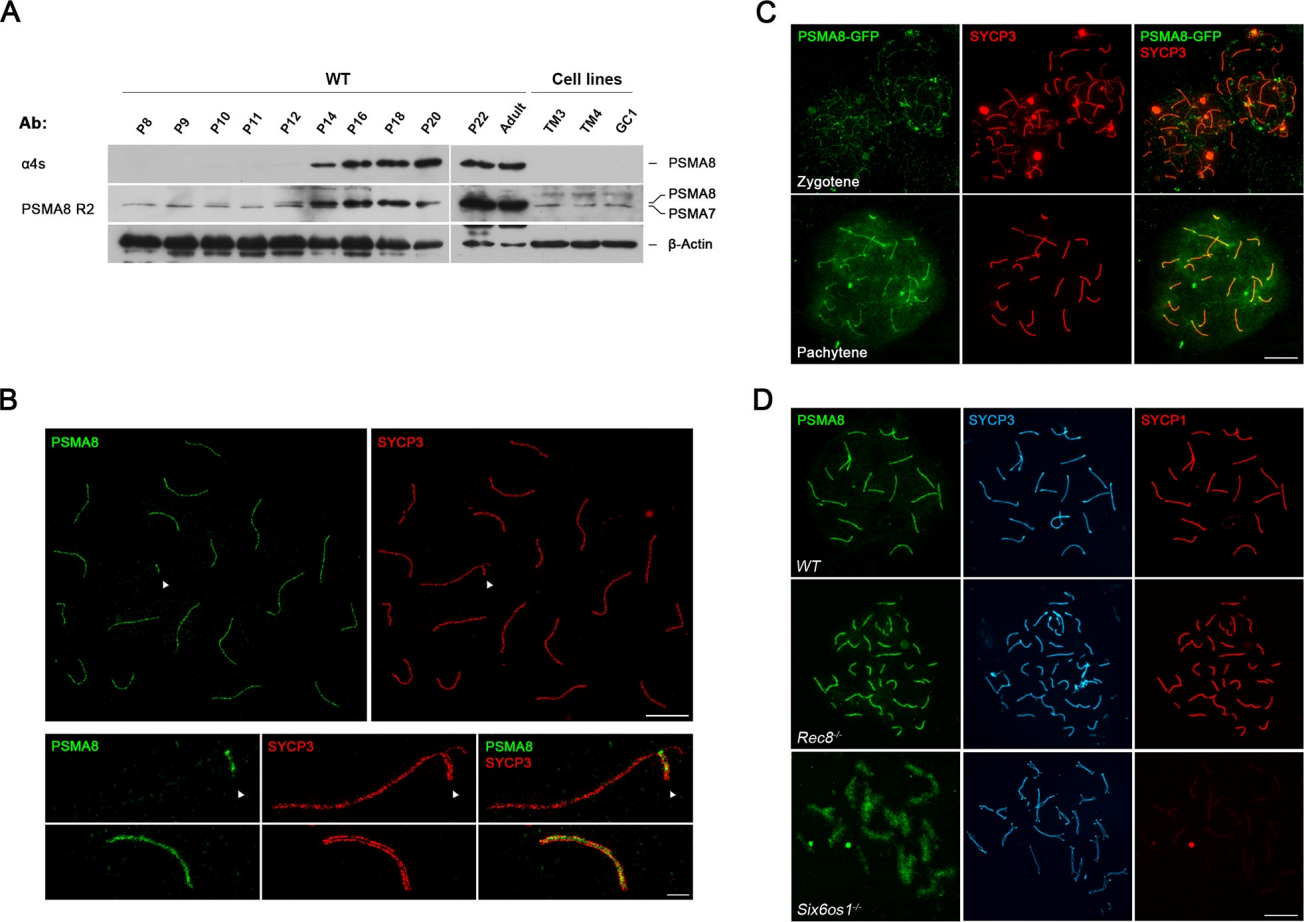
Expression analysis and localization of PSMA8 in the mouse. (A) Western blot analysis of protein extracts from mouse testis (from P8 to adult) and cell lines (TM3, TM4 and GC1) with a specific antibody against the C-terminal (α4S) and whole recombinant PSMA8 protein (PSMA8-R2). β-Actin was used as loading control. The corresponding bands to PSMA8 and PSMA7 are indicated in the right of the panel. Note that from P16 to adult the intensity of both PSMA8 and PSMA7 bands impedes its independent observation. (B) Double immunolabeling of spermatocyte spread preparations with PSMA8 (green) and SYCP3 (red) by Stimulated emission depletion (STED) microscopy, showing that PSMA8 localizes to the central region of the SC. PAR (pseudo-autosomal region) of the XY bivalent is indicated with an arrow. (C) Immuno-localization of PSMA8 in mouse testis after *in vivo* electroporation of a plasmid encoding a protein fusion of PSMA8 with GFP (GFP-PSMA8). PSMA8 was detected with anti-GFP antibody (green) and endogenous SYCP3 was detected using mouse anti-SYCP3 (red). (D) Triple labeling of PSMA8 (green), SYCP3 (blue) and SYCP1 (red) in *Rec8*^*-/-*^ and *Six6os1*^*-/-*^. PSMA8 is detected in the pseudosynapsed AEs of the meiotic *Rec8* cohesin mutant but is absent from the unsynapsed AEs in *Six6os1*^*-/-*^ spermatocytes. Bar in panels, 5 μm (B, upper panel), 1 μm (B, lower panel) and 10 μm (C, D).

*Psma8* mRNA expression in mouse tissues is almost exclusively restricted to the testis (GTEx database [23] and previous studies [10]). To elucidate the cell type in which PSMA8 is expressed, we examined by western blotting testis extracts at various postnatal ages during the first wave of spermatogenesis, which progresses more synchronously than in adult mice. PSMA8 expression (using a specific antibody against the PSMA8 C-terminus [10], see Fig 1A) was first detected at P12 and increased from P14 to P20. We also used a PSMA8-R2 antibody raised against the entire recombinant PSMA8 protein, which detected the expression of both PSMA7 (already apparent at P8, before meiosis has started) and PSMA8 (Fig 1A and S1 Fig). Analysis of testis cell lines (including spermatogonium GC1-spg, Leydig cell TM3, and Sertoli cell TM4 lines), revealed the expression of PSMA7 but not PSMA8 (Fig 1A). These results indicate that its expression is restricted to cells undergoing meiosis.

To explore the subcellular localization of PSMA8, we employed the R2 antibody (PSMA7/8) since the PSMA8 C-terminus antibody did not produce any specific labeling. Double immunolabeling of PSMA8 with the AE protein SYCP3 or with SYCP1, the transverse filament protein essential for synapsis (Fig 1B and S2 Fig), revealed PSMA7/8 presence at the central region of the synaptonemal complex (super resolution imaging, Fig 1B). We validated this localization by *in vivo* electroporating [24] an expression plasmid encoding GFP-PSMA8 in the testis (Fig 1C). These results agree with the recent localization of the proteasome to the chromosome axes [17].

To investigate the possible dependence of PSMA8 localization on synapsis, we analyzed synaptic mutants with mild (*Rec8*^*-/-*^ [25]) and severe (*Six6os1*^*-/-*^ [24]) phenotypes. Mutants for the meiotic cohesin REC8 show pseudo-synapsis between sister chromatids [25], and PSMA8 was detected at these atypical synapsed-like regions (Fig 1D). In mice lacking the novel central element protein SIX6OS1, in which AEs are physically separated and unsynapsed at pachynema [24], PSMA8 signal was not restricted to their AEs and showed a broader and more disperse labeling (Fig 1D). These results indicate that PSMA8 localization to the SC central region is consequently dependent on the assembly of the SC.

### Male mice lacking PSMA8 are infertile

To study the role of PSMA8, we generated a targeted mutation in exon 1-intron 1 of the murine *Psma8* gene by CRISPR/Cas9 genome editing (S3A and S3B Fig). Homozygous mutant testes showed no PSMA8 protein expression by western blotting when analyzed using two independent polyclonal antibodies (S3C Fig). Immunofluorescence analysis of PSMA8 expression (R2 antibody, S3D Fig) revealed a weaker signal in the SC of the mutant spermatocytes than in WT spermatocytes (51% less; 4.22±1.9 WT vs 2.05±1.7 KO), likely representing PSMA7 detected by the R2 antibody (also observed in the western blot; S3C Fig). These results indicate that the generated mutation is a null allele of the *Psma8* gene (herein termed *Psma8*^-/-^).

**Fig 2:**
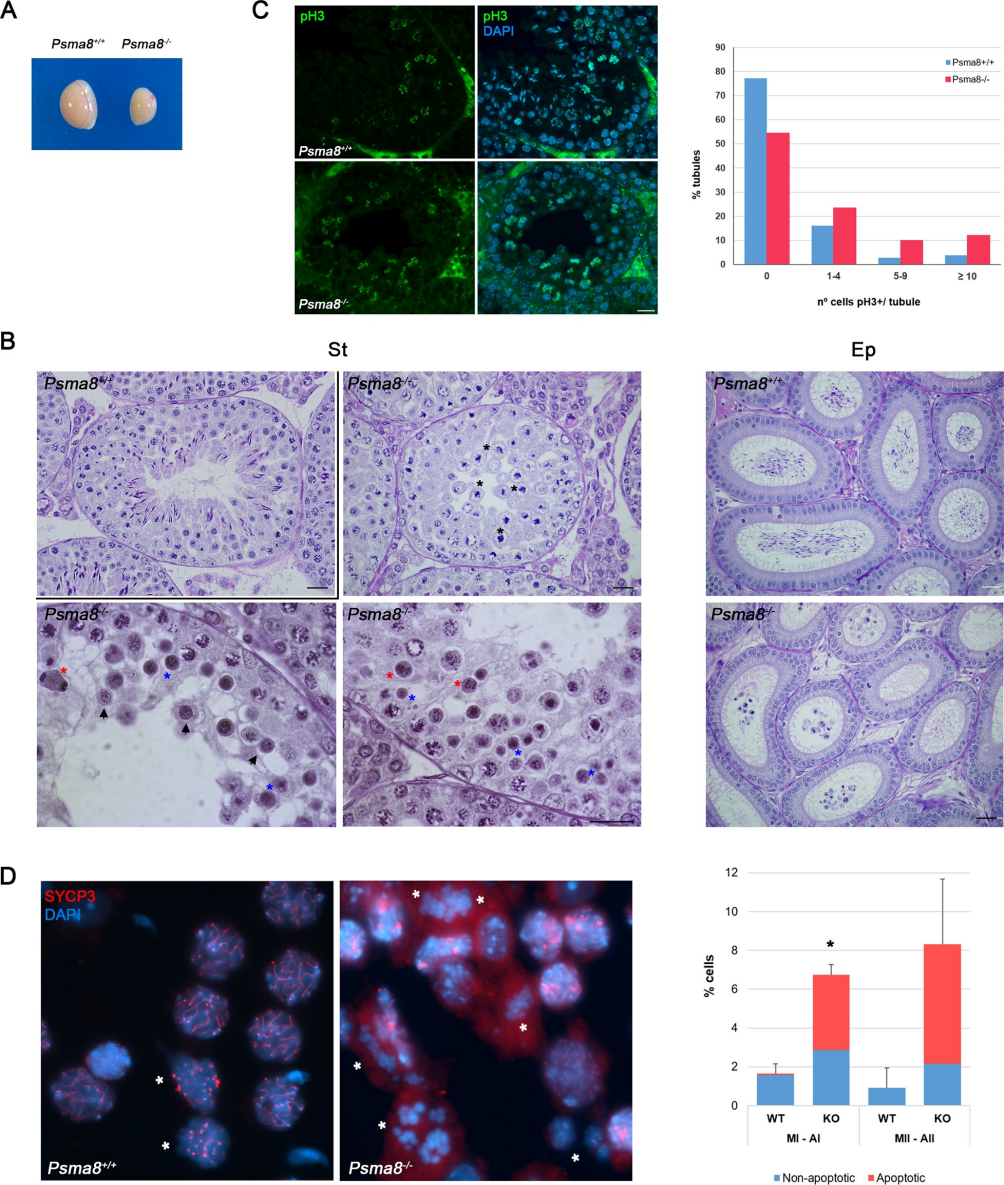
PSMA8 deficiency leads to azoospermia. (A-B) Genetic ablation of *Psma8* leads to a reduction of the testis size (A) (n = 6, WT and KO; Welch´s *t*-test analysis: p<0.0001), and (B) the accumulation of metaphase I (black asterisks), apoptotic meiotic division (red asterisks), round spermatids entering apoptosis (arrowheads), and apoptotic round spermatids (blue asterisks) in PAS stained testis sections. The spermatogenic arrest leads to empty epididymides and azoospermia. Bar in upper panels 100 μm, lower panels 200 μm and in right panels, 5 μm. (**St**) Seminiferous tubules and (**Ep**) Epididymides. (C) Immunofluorescence analysis of p-ser10-H3 (green) of paraffin sections of *Psma8*^*+/+*^ and *Psma8*^*-/-*^ tubules. Nuclei were counterstained with DAPI. Bar represents 10 μm. The diagram represents the quantification of the fraction of tubules showing the indicated number of metaphase I/II. Number of tubules counted for each genotype is expressed in S2B Table. (D) Low magnification view of a representative squash preparation of seminiferous tubules showing the accumulation of metaphases I and metaphases II in knock-out *Psma8* in comparison with a representative wild-type view. The identity of metaphases I /metaphases II (asterisks) was confirmed by the immunolabeling of SYCP3 (red) in squash preparations. Chromosomes were counterstained with DAPI (blue). The diagram represents the percentage of spermatocytes at metaphase I and II (normal and apoptotic) in relation with the total number of spermatocytes from *Psma8*^*+/+*^ and *Psma8*^*-/-*^ tubules (right). Quantification and number of cells analyzed are described in S2C Table. Welch´s *t*-test analysis: * p<0.01; ** p<0.001; *** p<0.0001.

**Fig 3:**
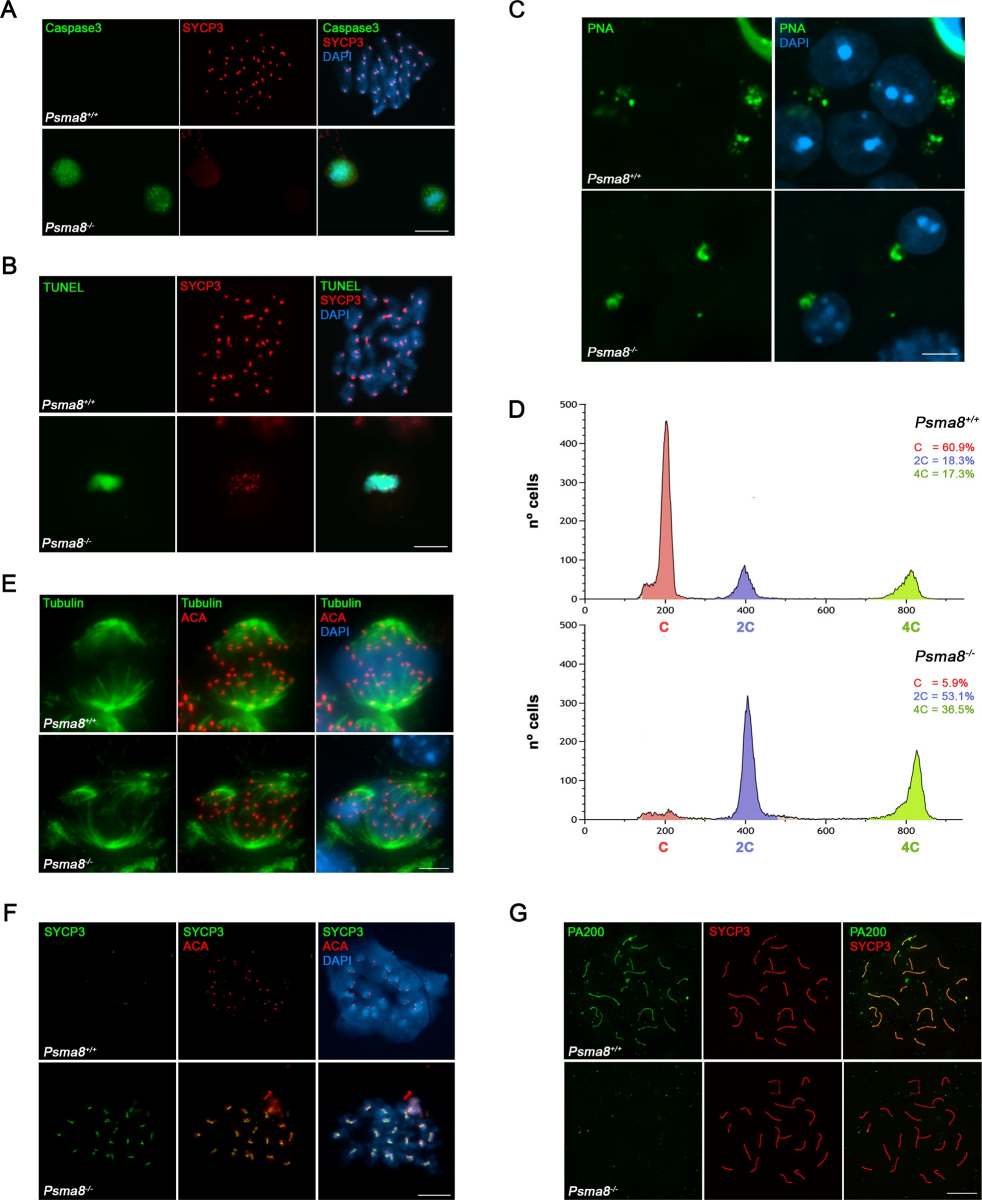
Apoptosis, FACs and aberrant metaphase II and spermatid cells in *Psma8*-deficient mice. (A) Double immunolabeling of Caspase3 (green) and (B) TUNEL (green) with SYCP3 (red). Non-apoptotic metaphase I cells from *Psma8*^+/+^ show absence of green staining whereas apoptotic metaphases I from *Psma8*^-/-^ show intense Caspase-3 and TUNEL labeling. Chromatin was counterstained with DAPI. (C) Acrosome positive labeling of round spermatids by PNA staining (green). (D) FACs analysis of cells from whole seminiferous tubules from wild type and *Psma8*^*-/-*^ showing in both genotypes (N = 2) the presence of 4C, 2C and 1C compartment as a result of the early spermatid arrest. Source data describing the gating strategy is shown in S5B Fig. (E) Double immunolabeling of metaphase I cells with tubulin (green) and ACA (red) showing normal (*Psma8*^*+/+*^) and abnormal spindles (*Psma8*^*-/-*^). (F) Double immunolabeling of SYCP3 (green) with ACA (red) in wild-type and *Psma8*^*-/-*^ spermatocytes at metaphase II which shows aberrant accumulation of SYCP3 at the centromeres. (G) Double immunolabeling of PA200 (green) and SYCP3 (red) in chromosome spreads. PA200 is detected at the chromosome axes of the autosomal and XY bivalents during pachytene in wild type spermatocytes in contrast to the absence of labeling in *Psma8*^*-/-*^ spermatocytes. Bar in panels (C, E) 5 μm and 10 μm (A, B, F and G).

Mice lacking PSMA8 did not display any somatic abnormalities; however, male but not female mice were sterile (S1 Table). Indeed, *Psma8* mutation resulted in a reduction of the testis weight (63.09% decrease; N = 6) and the absence of spermatozoa in the epididymides (Fig 2A and 2B). Histological analysis of adult *Psma8*^-/-^ testes revealed the presence of apparently normal numbers of spermatogonia, spermatocytes, Sertoli cells and Leydig cells (Fig 2B). Mouse seminiferous tubules can be classified from epithelial stage I to XII by determining the groups of associated germ cell types in histological sections. Following these criteria, we found that spermatogenesis in the mutant testes proceeded normally up to diplotene in epithelial stage XI. However, the proportion of tubules at stage XII was more than 2-fold increased in the mutant sections (12.5% in mutants versus 5.4% in WT, S2A Table). Given that spermatocytes in meiotic divisions were seen to occur at epithelial stage XII, we used p-ser10-H3 (pH3) staining to analyze the number of metaphase I and II cells present in these tubules, finding an increase in the mutant (Fig 2C and S2B Table). Quantitative analysis of seminiferous tubules in squashed preparations confirmed the increase in the number of metaphase I and metaphase II cells as compared with WT testes (77% and 89% respectively, Fig 2D and S2C Table). Moreover, a large proportion of these metaphases were positive for Caspase-3 and TUNEL indicating apoptosis (Figs 2D, 3A and 3B and S2C Table).

Together with the accumulation of apoptotic meiotic divisions, other apoptotic cells could be also observed that, from their size and molecular markers of the acrosome and chromatoid body, were round spermatids (Fig 3C and S4 Fig). Indeed, seminiferous tubules in PSMA8-deficient testes sometimes contained a few surviving round spermatids. However, these round spermatids were unable to form a proper acrosome but did accumulate some PAS positive material. Apoptotic round spermatids were also seen and no elongating spermatids were observed (Fig 2B). We corroborated that round spermatids were arrested at early stages by immunolabeling for H2AL2. H2AL2 is a transition histone essential for the first replacement of histones by TNP1 and TNP2 before protamine incorporation [26]. H2AL2 was absent from mutant spermatids (S5A Fig). We also used FACs analysis of whole cells from seminiferous tubules to verify this analysis. The results obtained confirmed the presence of a small haploid compartment in *Psma8*^*-/-*^ testes (Fig 3D and S5B Fig). We conclude from these results that PSMA8 deficiency causes the accumulation of spermatocytes in metaphase I and II which either enter massively into apoptosis or give rise to a low number of aberrant round spermatids that finally apoptose long before histone replacement takes place.

### *Psma8*-deficient spermatocytes show normal synapsis/desynapsis and DSB repair but have abnormal metaphases I and II

Metaphase I accumulation can occur either because of a failure to enter anaphase or because of some event taking place during prophase (SC formation, DBSs repair or chromosome recombination) that aberrantly triggers a checkpoint-mediated delay.

To test this, we first analyzed the assembly/disassembly of the SC by monitoring the distribution of SYCP1, as co-labeling of SYCP3 and SYCP1 highlights regions of synapsis in spermatocytes. We did not observe any differences in this process from zygonema to diakinesis (S6 Fig).

We next studied the kinetics of DSB repair during meiosis. Meiotic DSBs are generated by the nuclease SPO11 and are then resected to form ssDNA ends that invade into the homologous chromosome. DSBs are marked by the presence of phosphorylated H2AX (γ-H2AX) [27]. The distribution of γ-H2AX in mutant spermatocytes was similar to that found in WT cells at prophase I (S7A Fig and S3 Table). We also did not observe any differences in the distribution of RAD51, a recombinase that promotes homologous strand invasion [28], (S7B Fig and S3 Table). Because defective DNA repair ultimately abrogates CO formation [29] and because of the involvement of ubiquitylation / sumoylation in CO designation [30], we analyzed the distribution of MLH1 foci [31], a mismatch repair protein (marker of crossover sites) that functions in the resolution of joint molecules at the end of crossover formation [32]. We found a similar value between the KO (24.9±0.9 foci) and the WT (24.3±1.1 foci; S7C Fig and S3 Table). These results indicate that the repair of meiotic DSBs and synapsis/desynapsis proceed normally during prophase I in the absence of PSMA8, and is not responsible for the observed metaphase I accumulation.

We also analyzed the morphology of the metaphase I / II cells by staining for tubulin (spindle) and SYCP3. The results showed an aberrant morphology, the presence of multipolar spindles (Fig 3E), and also a striking aberrant labeling of SYCP3 at the centromeres of metaphase II chromosomes (SYCP3 labeling is barely visible in metaphase II sister kinetochores in WT cells, Fig 3F). Finally, the arrested round spermatids showed the presence of multiple patches of heterochromatin after DAPI staining (Fig 3C and S4 Fig, chromocenter fragmentation), suggesting abnormal chromosome segregation or cytokinesis.

### PSMA8 deficiency abolishes H4ac turnover from late prophase to round spermatids

During spermiogenesis, most of the histones are replaced by basic transition proteins, and ultimately by protamines, facilitating chromatin compaction. Hyperacetylation of core histones during this process, and especially the acetylation of H4K16, is assumed to play a pivotal role in the initiation of histone displacement and chromatin ultracondensation [33, 34]. The proteasome activator subunit PA200 targets acetylated histones for degradation during histone replacement [5].

The core subunit PSMA8 co-immunoprecipitated PA200 (S4 Table). Given the stoichiometric relationship between the CP and RP, we analyzed the expression of PA200 by immunofluorescence in the absence of PSMA8. Whilst PA200 decorated the AEs of WT spermatocytes, we failed to observe any signal in the AEs of mutants (Fig 3G and S8 Fig). In addition, we were not able to detect PA200 by mass spectrometry analysis of PSMA7/8 immunoprecipitation of *Psma8*-deficient testis extracts (see section Purification of PSMA8-interacting proteins, S4 Table). These results indicate that PSMA8 is necessary or promotes the assembly of PA200 to the CP. Thus, within the limits of detection, the deficiency of *Psma8* leads to a drastic decrease of PA200.

**Fig 4:**
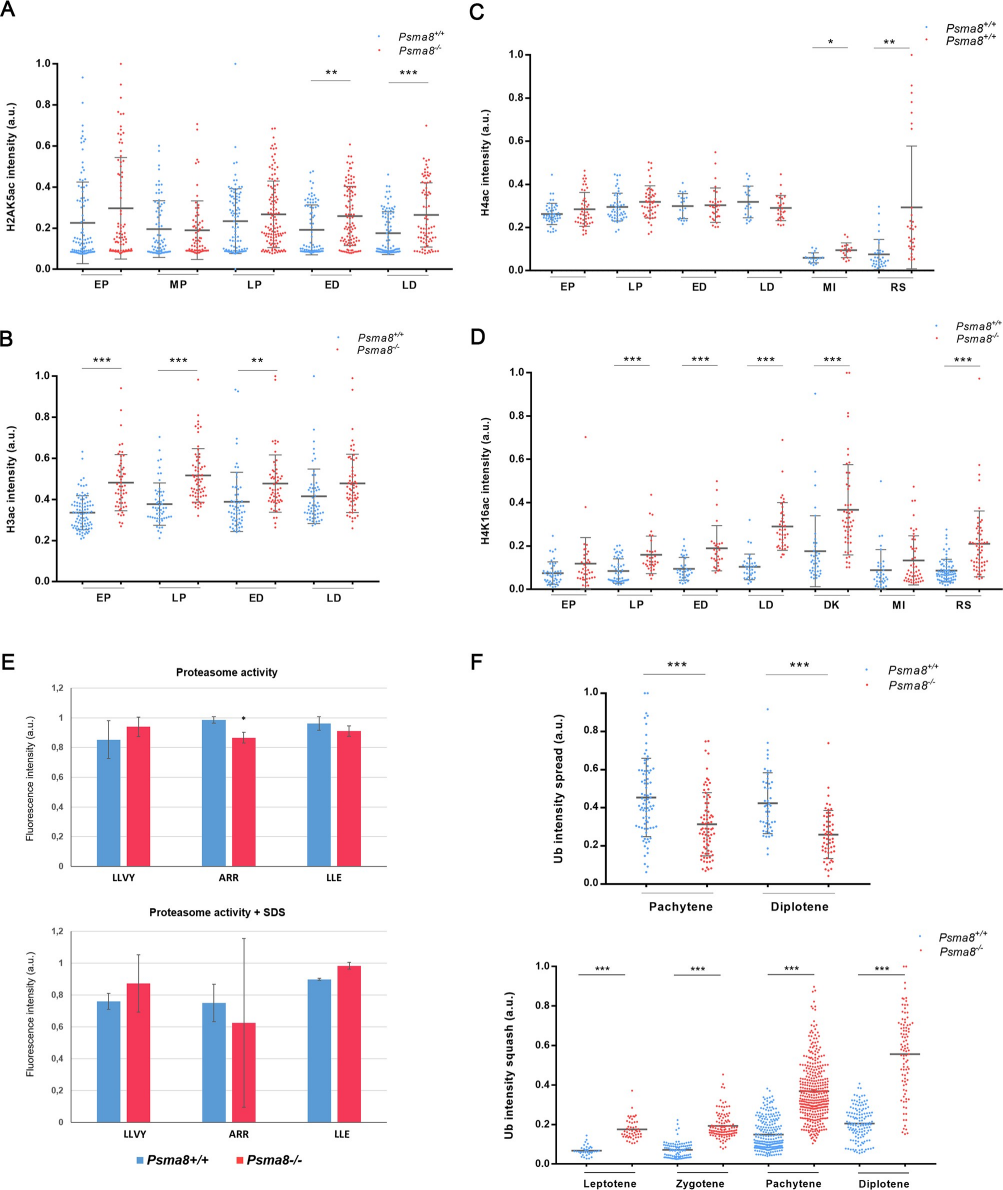
Histone acetylation, nuclei ubiquitylation and proteasome activity in PSMA8-deficient mice. (A-D) Plots represent the quantification of the fluorescence intensity from *Psma8*^*+/+*^ and *Psma8*^*-/-*^ spermatocytes at early pachytene (EP), mid pachytene (MP), late pachytene (LP), early diplotene (ED), late diplotene (LD), diakinesis (DK), metaphase I (MI) and round spermatid (RS) corresponding to the immunolabeling of (A) H2AK5ac, (B) H3ac, (C) H4ac, and (D) H4K16ac. Representative figures for each immunofluorescence are presented in S9–S12 Figs. (E) Proteasome activity of *Psma8-*deficient testis. 100 μg of protein from whole testis extracts of *Psma8*^*+/+*^and *Psma8*^*−/−*^ mice were inoculated into 96-well plate and the proteasome peptidases activities were measured. The enzymatic activities relative to WT are shown. (F) Plots represent the quantification of the fluorescence intensity from *Psma8*^*+/+*^ and *Psma8*^*-/-*^ spread (upper) and squashed (lower) spermatocytes. Welch´s *t*-test analysis: * p<0.01; ** p<0.001; *** p<0.0001.

To understand the acetylated-dependent degradation of histones by the proteasome [5], we measured the acetylation status of three core histones, H2AK5ac, H3ac and H4ac (pan-H4ac and H4K16ac) in chromosome spreads by double immunolabeling for SYCP3 and the corresponding acetylated histone (Fig 4A–4D and S9–S12 Figs). This procedure enables a more precise staging of the spermatocytes and is a more efficient mean to quantitate signals than peroxidase immunostaining of testis sections [5]. The loss of PSMA8 led to the accumulation of H2AK5ac, H3ac, H4ac and H4K16ac, albeit to different degrees. Results showed that the levels of H2AK5ac, H3ac, H4ac and H4K16ac were moderately higher in *Psma8*^*-/-*^ cells, with a relative increase at late prophase I (Fig 4A–4D and S9–S12 Figs). We failed to detect staining for H2AK5ac and H3ac in spermatocytes in late diakinesis and round/arrested spermatids. In contrast, pan-H4ac and H4K16ac also labeled metaphase I chromosomes, interkinesis nuclei and round/arrested spermatids, with greater intensity in mutant than in WT cells (Fig 4C and 4D and S11 and S12 Figs). The accumulation of acetylated histones during prophase I and particularly of H4ac and H4K16ac in the arrested round spermatids suggests that the PSMA8-containing proteasomes are involved in the acetylation-dependent degradation of histones.

### Proteasomal activity in *Psma8*-deficient mice

We next investigated the biochemical activity of testis extracts lacking PSMA8-containing proteasomes by measuring chymotrypsin-like activity (corresponding to the catalytic subunit β1), caspase-like activity (corresponding to β5) and trypsin-like activity (β3) by a standard fluorogenic assay [35] in the presence and absence of SDS (activated proteasome). Results showed that proteasomal activity in *Psma8*-deficient testis extracts was not noticeably different from that in WT extracts. Indeed, the trypsin-like activity was the only proteolytic function with a modest reduction in the KO (Fig 4E). Overall, these results show that the general proteasome activity of the *Psma8*-deficient testis is not radically changed, which is likely due to the presence of PSMA7-dependent CPs (see dataset 1 in [36]).

To ascertain the degree of activity *in vivo*, we first investigated the steady-state levels of protein ubiquitylation in testis during mouse meiosis. Using immunofluorescence, we analyzed spermatocytes obtained from spreads and squashed preparations with ubiquitin antibodies (Fig 4F and S13 Fig). The results showed a slight decrease of chromatin bound ubiquitylated proteins but an increase in the soluble fraction of ubiquitylated proteins during prophase I (Fig 4F and S13 Fig). These results are partially in agreement with the observed increase in the ubiquitylation state of cultured spermatocytes treated with the proteasome inhibitor MG132 (18), and suggest a specific function of the PSMA8-containing proteasomes in the controlled degradation of ubiquitylated proteins during spermatogenesis.

### Purification of PSMA8-interacting proteins

The composition of the CP and its RPs has previously been established by mass-spectrometric analysis of crude preparation of proteasomes from whole testes [37]. To better understand the molecular mechanism underlying the mutant phenotype, we purified PSMA7/8-interacting proteins by single-step affinity chromatography (see Material and methods for a detailed description). Most of the canonical subunits of the CP and RP were present within the more than 596 proteins of the PSMA8 proteome (S5 Table, using a conservative cut-off, see methods). In agreement with previous results, among the two activators of the testis-specific proteasome detected (PA200 and Pa28γ) [5], PA200 was the most abundant. In contrast to previous observations, we were unable to detect Pa28α and Pa28β or the inducible catalytic subunits of the immunoproteasome (β1i, β2i and β5i) [5], suggesting a very low abundance or absence. We could not detect PA200 as an interacting protein of PSMA7/8 in testis extracts from *Psma8*-deficient testes (S4 Table).

Among the novel proteasome-interacting proteins (PIPs) detected were chaperones including CCT6b and CCT2, ubiquitin ligases (TRIP12, NEDD4, TRIM36 and RAD18), and novel ubiquitin specific proteases (USPs) such as USP9X, USP34, USP5 and USP47 (S6 Table). We studied the proteins enriched in the immunoprecipitation through functional (gene ontology, GO) and pathway analysis (KEGG). The top GO and KEGG results were related to the proteasome and to ribonucleoproteins. Pathway analysis showed links to spermatogenesis, cell cycle, and meiosis (see S1 Text), in accordance with the observed mutant phenotype.

Interestingly, we identified meiotic proteins *a priori* unrelated to the UPS such as DAZL (deleted in azoospermia), SPAG1 (Sperm-associated antigen 1), SPATA5/20 (Spermatogenesis-associated protein 5/20), the tudor domain proteins TDRD1/6/9, MAEL (repressor of transposable elements), and RNF17. These PIPs could represent proteins captured during ubiquitin-dependent targeted degradation [38] and/or proteins interacting *via* ubiquitin-independent proteasomal degradation, as has been shown for the related subunit α4/PSMA7 [39]. Altogether, the list of novel PIPs included novel potential readers, erasers and writers of the ubiquitin code [40] of the testis-specific proteasome, reflecting its complexity. Among these PIPs, we focused our attention on the following candidates for their role in chromosome segregation and synapsis: SYCP1, TRIP13, TEX30, PIWIL1, PIWIL2 and CDK1 (S6 Table).

**Fig 5:**
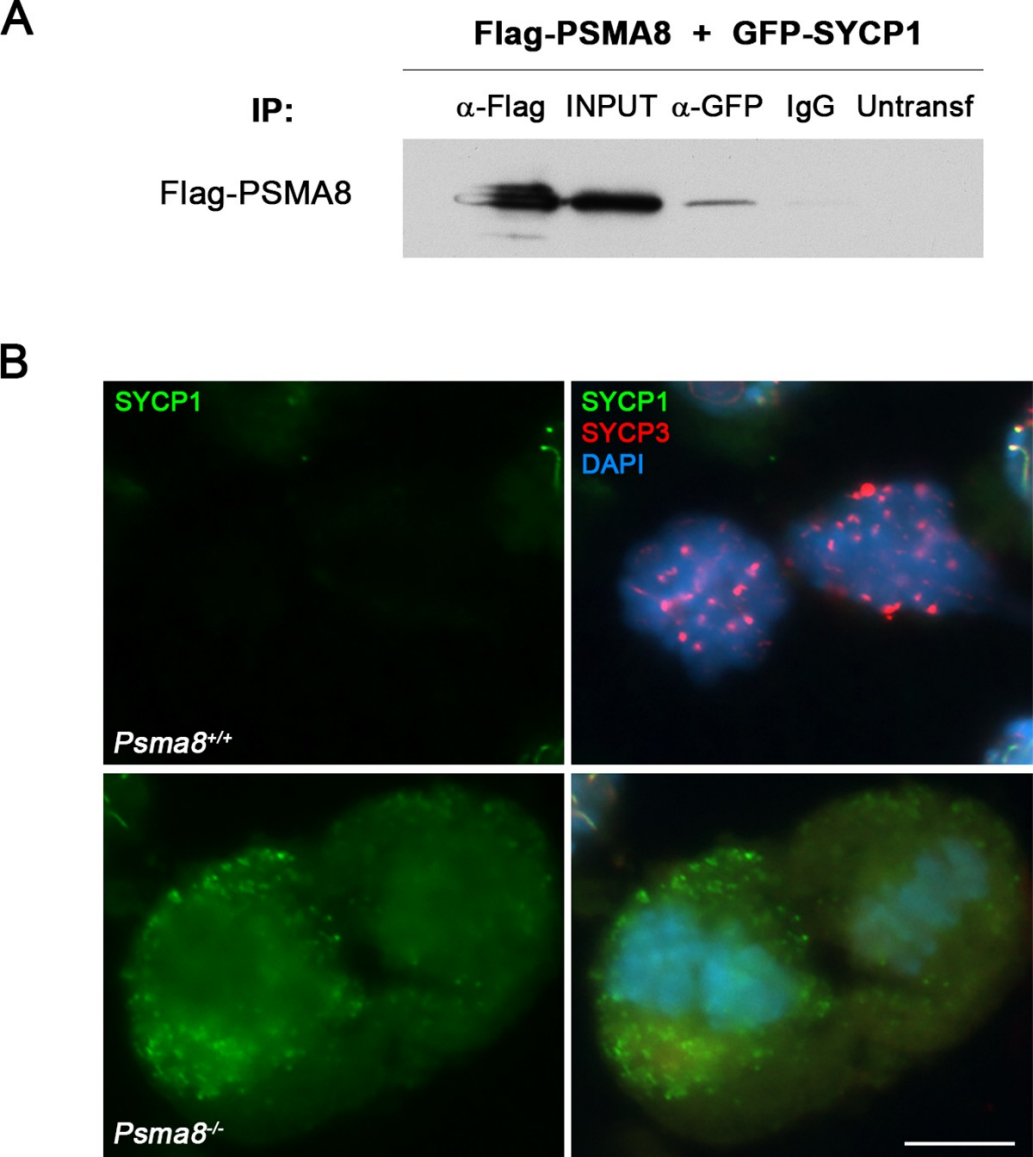
SYCP1 interacts with PSMA8 and is accumulated in *Psma8*-deficient metaphase I cells. (A) HEK293T cells were transfected with Flag-PSMA8 and GFP-SYCP1. Protein complexes were immunoprecipitated overnight with either an anti-Flag or anti-EGFP or IgGs (negative control), and were analyzed by immunoblotting with the indicated antibody. PSMA8 co-immunoprecipitates with SYCP1. (B) Double immunolabeling of squashed tubules with SYCP1 (green) and SYCP3 (red) in wild-type and *Psma8*^*-/-*^ spermatocytes at metaphase I. Chromatin was stained with DAPI (blue). Bar in panel, 10 μm.

Among the possible interactors, we first evaluated the transverse filament protein SYCP1. Because *Sycp1* mutant mice are infertile but otherwise healthy [41], we analyzed the interaction of SYCP1 with PSMA8 and its localization in mutant meiosis. We co-transfected *Sycp1* with *Psma8* in HEK293T cells and we detected co-immunoprecipitation between SYCP1 and PSMA8 (Fig 5A). Despite the observation that SYCP1 is properly loaded to the SC and removed from desynapsed regions (S6 Fig), we observed an abnormal accumulation of SYCP1 in *Psma8*-deficient metaphase I cells, (Fig 5B). These results suggest defective degradation of SYCP1 with very likely detrimental functional consequences in the exit of meiosis.

**Fig 6:**
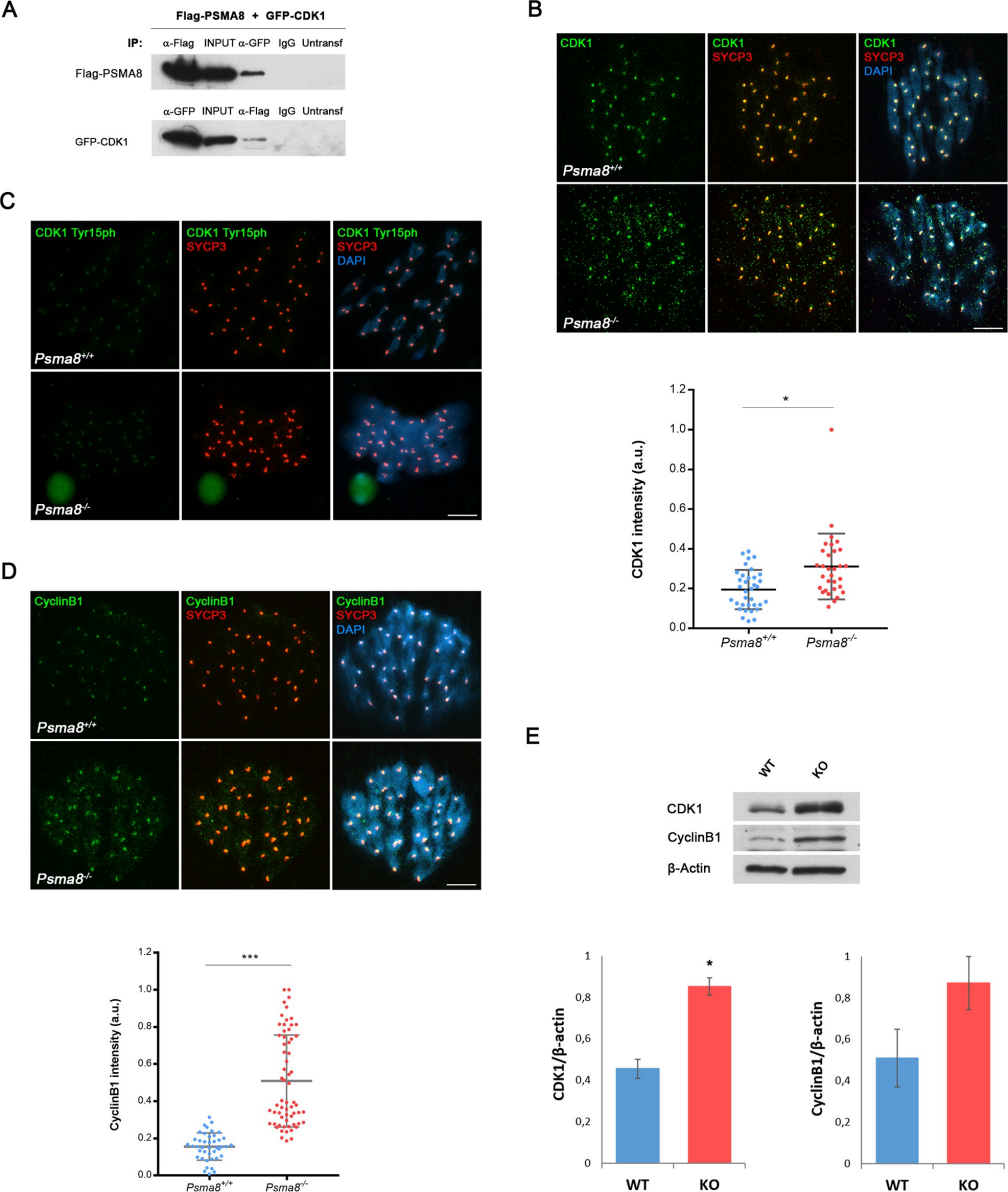
PSMA8 deficiency causes an accumulation of CDK1 and Cyclin B1 in spermatocytes. (A) HEK293T cells were transfected with Flag-PSMA8 and GFP-CDK1. Protein complexes were immunoprecipitated with either an anti-Flag or anti-EGFP or IgGs (negative control) and were analyzed by immunoblotting with the indicated antibody. PSMA8 co-immunoprecipitates with CDK1 (as well as reciprocally). (B) Double labeling of endogenous CDK1 (green) and SYCP3 (red) in mouse spermatocytes at metaphase I. Chromatin was stained with DAPI (blue). During metaphase I, CDK1 labels in a slight and disperse way the chromosomes and in a more intensely fashion the centromeres of bivalents. This labeling pattern is enhanced in a normal *Psma8*-deficient metaphase I. Plot under the panel represents the quantification of the fluorescence intensity from *Psma8*^*+/+*^ and *Psma8*^*-/-*^ metaphase I cells. (C) Double labeling of endogenous CDK1-Tyr15phosphorylated (green) and SYCP3 (red) in mouse spermatocytes at metaphase I showing similar expression levels in *Psma8*^*+/+*^ and *Psma8*^*-/-*^. Chromatin was stained with DAPI (blue). (D) Double labeling of endogenous cyclin B1 (green) and SYCP3 (red) in mouse spermatocytes at metaphase I showing higher expression levels in *Psma8*^*-/-*^. Plot under the panel represents the quantification of the fluorescence intensity from *Psma8*^*+/+*^ and *Psma8*^*-/-*^ metaphase I cells. Welch´s *t*-test analysis: * p<0.01; ** p<0.001; *** p<0.0001. (E) CDK1 and CyclinB1 were measured by western blot analysis of protein extracts from whole testis of *Psma8*^*+/+*^ (WT) and *Psma8*^*-/-*^ (KO) (n = 2 mice). Bar in panels, 10 μm. Welch´s *t*-test analysis: * p<0.05; ** p<0.001; *** p<0.0001.

**Fig 7:**
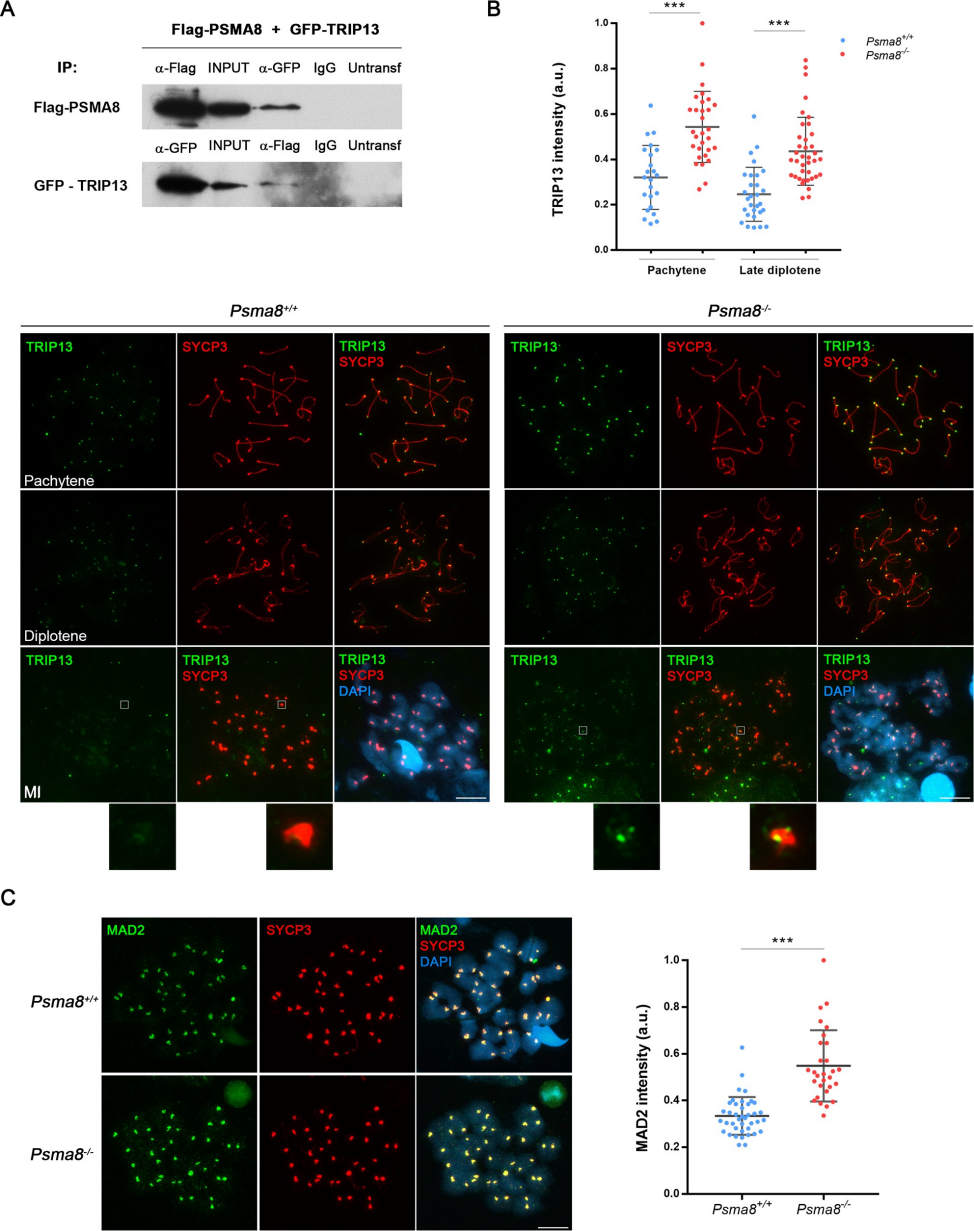
TRIP13 and MAD2 levels are increased in *Psma8*-deficient spermatocytes. (A) HEK293T cells were transfected with a plasmid encoding GFP-TRIP13 and Flag-PSMA8. Protein complexes were immunoprecipitated with either an anti-Flag or anti-EGFP or IgGs (negative control), and immunoblotted with the indicated antibody. (B) Double immunolabeling of TRIP13 (green) and SYCP3 (red). TRIP13 labels the telomeres at pachytene and the intensity of the labeling decreases through desynapsis at diplotene and diakinesis. This labeling is enhanced during prophase I in the *Psma8* mutants but its main pattern is not altered. At metaphase I, a faint labeling of sister kinetochores is observed in the *Psma8*^*-/-*^ spermatocytes that is absent in the wild type. Plot over the panel represents the quantification of the fluorescence intensity from *Psma8*^*+/+*^ and *Psma8*^*-/-*^ spermatocytes at pachytene and late diplotene. (C) MAD2 (green) labels with enhanced intensity the centromeres of the chromosomes from *Psma8*^*-/-*^ metaphase I cells in comparison with the WT controls. Plot right to the panel represents the quantification of the fluorescence intensity from *Psma8*^*+/+*^ and *Psma8*^*-/-*^ spermatocytes at metaphase I spermatocytes. Bar in panels, 10 μm. Welch´s *t*-test analysis: * p<0.01; ** p<0.001; *** p<0.0001.

We next extended the validation analysis of the remaining candidate interactors by co-immunoprecipitation with PSMA8, making use of the same heterologous system of HEK293T cells. These included TEX30, PIWIL1, PIWIL2, CDK1 and TRIP13. All protein-protein interaction assays carried out were negative (S14A Fig) with the exceptions of the cyclin dependent kinase CDK1 and the AAA-ATPase TRIP13 (AAA-ATPases associated with diverse cellular activities; see Figs 6A and 7A). Because of the relevance of CDK1 in metaphase transition, we first determined the expression levels of CDK1 by immunofluorescence. The results showed that more CDK1 but not the related kinase CDK2 [42] could be detected in the centromeres of metaphase I chromosome from mutant cells (Fig 6B and S15A Fig; KO 0.31±0.2 vs 0.19±0.1 WT; an increase of ~ 40%). To determine whether the increased level of CDK1 corresponded to its active or inactive phosphorylated form, we used an antibody against CDK1-Tyr15-p (inactive form, Fig 6C). The results showed no differences in the labeling at the centromeres of the metaphase I chromosomes, and therefore a decrease in phospho-CDK1/total CDK1 ratio in mutant cells. Given that CDK1 must be complexed with cyclin B1 to be active, we reasoned that if higher levels of active CDK1 are present, cyclin B1 would be similarly increased. Results showed an increase of cyclin B1 at the centromeres of metaphase I chromosomes (Fig 6D). This result was congruent with the increased amount of CDK1 and CyclinB1 observed by western blot and in squashed seminiferous tubules (Fig 6E and S15B and S15C Fig). Overall, these findings suggest that loss of PSMA8 causes an increase of CDK1 / CyclinB1 which would cooperate in the accumulation of metaphase I / metaphase II that ultimately results in apoptotic metaphase plates.

We also analyzed the distribution of TRIP13, a pleiotropic ATPase that participates in meiotic DNA repair and chromosome synapsis through HORMAD interaction and somatic spindle assembly checkpoint (SAC) proficiency through MAD2 interaction [43–46]. We first performed immunofluorescence analysis of TRIP13 in *Psma8-*deficient and WT spermatocytes. Results using two independent antibodies showed robust labeling of the telomeres from zygonema (two dots) to pachynema (fused to a single dot) in WT cells, which declined from diplonema to diakinesis. The staining pattern was similar but enhanced in mutant spermatocytes (Fig 7B). However, the staining pattern of TRIP13 at metaphase I differed between WT and mutant cells. Specifically, it was detected at the kinetochores of *Psma8*^-/-^ spermatocytes but was absent in WT cells (Fig 7B). This labeling pattern at the metaphase I kinetochores resembles TRIP13 staining in somatic cells [47]. These results thus suggest that TRIP13 accumulates in the absence of a functional PSMA8-containing proteasome.

We next analyzed several downstream effectors of TRIP13, HORMAD1, HORMAD2, and the mitotic checkpoint protein MAD2 [48–50]. No differences were observed in the HORMAD1/2 labeling pattern between WT and mutant cells (S16 Fig). It has been shown in *C*. *elegans* that in the absence of TRIP13, MAD2 recruitment to kinetochores is delayed and that in addition to its role in checkpoint silencing, TRIP13 also contributes to spindle checkpoint activation [50]. It could thus be argued that an excess of TRIP13 would increase MAD2 loading to kinetochores thereby delaying mitotic exit. We confirmed this prediction and found that MAD2 expression at the kinetochores was enhanced in *Psma8*^-/-^ spermatocytes (Fig 7C), further validating a functional consequence of TRIP13 accumulation at the kinetochores.

In order to validate the substrate specificity of the PSMA8-containing proteasome in protein degradation, we analyzed the expression levels of the separase inhibitor securin (PTTG1), a well-known substrate of the somatic proteasome. Immunofluorescence analysis showed similar levels of PTTG1 in *Psma8*^*-/-*^ and WT spermatocytes (S17 Fig). This result suggests that PSMA8-containing proteasomes are not involved in the degradation of classical ubiquitylated substrates degraded by the somatic proteasome.

### PSMA8 interacts with proteins of the synaptonemal complex

**Fig 8:**
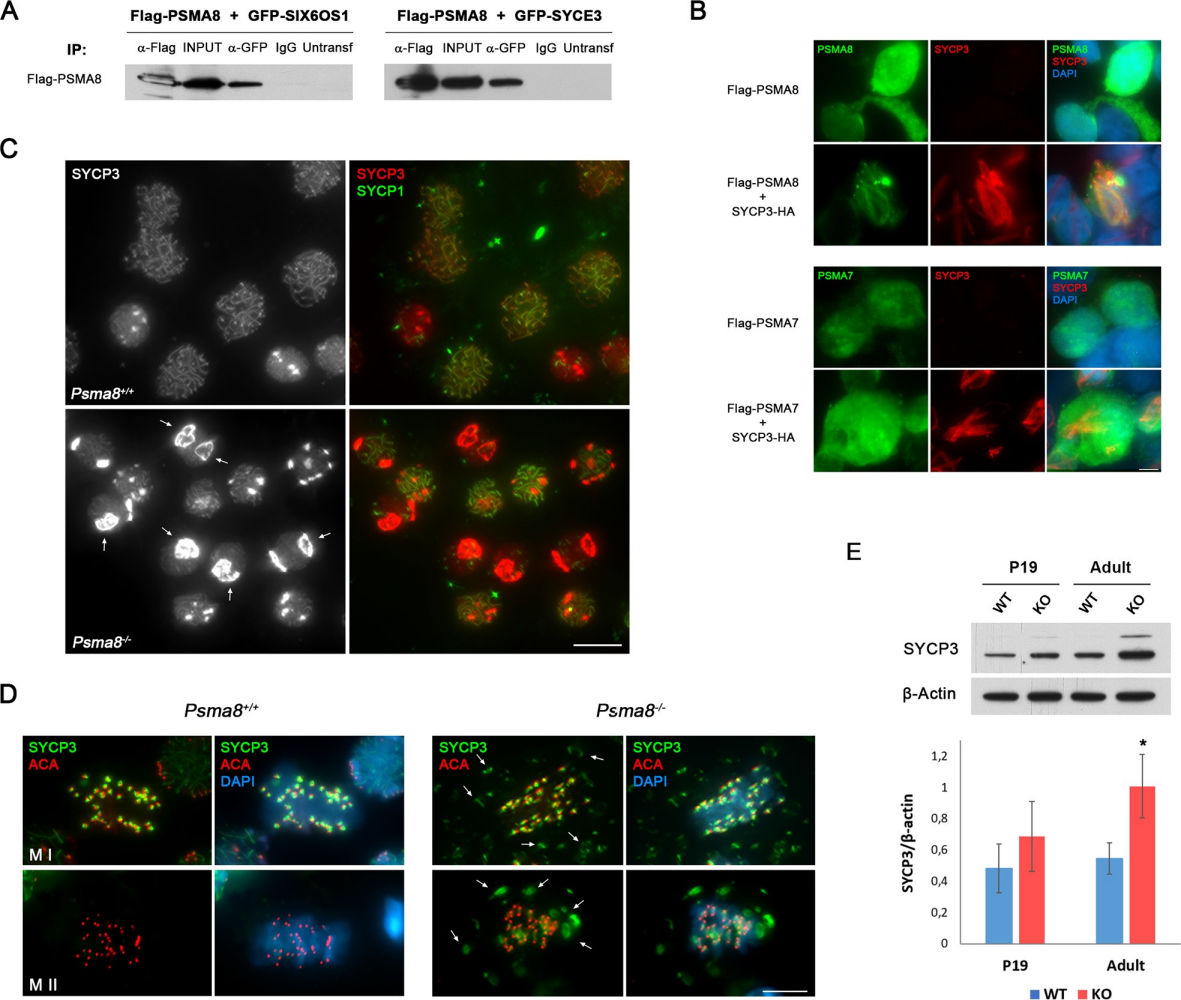
PSMA8 interacts with proteins of the SC. (A) PSMA8 co-immunoprecipitates with SIX6OS1 and SYCE3. HEK293T cells were transfected with plasmids encoding Flag-PSMA8 and GFP-SIX6OS1 or GFP-SYCE3. Protein complexes were immunoprecipitated overnight with either an anti-Flag or anti-EGFP or IgGs (negative control), and were analyzed by immunoblotting with the indicated antibody. (B) Double immunofluorescence of transfected HEK293T cells with plasmids encoding Flag-PSMA8 and Flag-PSMA7 alone or together with plasmid encoding SYCP3-HA and immuno-detected with antibodies against Flag (green) or HA (red). Transfected PSMA8 alone is delocalized and occupies the whole cell whereas when co-transfected with SYCP3-HA is recruited to form polycomplexes. PSMA7 do not form polycomplexes when co-transfected with SYCP3-HA. (C-D) SYCP3 is accumulated *in vivo* in *Psma8*^*-/-*^ spermatocytes. (C) Double immunolabeling of squashed tubules with SYCP3 (red) and SYCP1 (green) in wild-type and *Psma8*^*-/-*^ spermatocytes at prophase I showing large SYCP3 aggregates surrounding the nuclei (arrows). (D) Double immunolabeling of squashed tubules with SYCP3 (green) and ACA (red) in wild-type and *Psma8*^*-/-*^ spermatocytes at metaphase I and II. *Psma8*^*-/-*^ metaphases I show labeling of SYCP3 in aggregates (arrows, absent in the WT) in addition to its typical labeling at the centromeres. Metaphases II from *Psma8*^*-/-*^ show labeling for SYCP3 at the centromeres between the sister kinetochores and as aggregates in the cytosol (arrows) whereas wild type metaphases II show barely visible SYCP3 labeling. (E) SYCP3 was measured by western blot analysis of protein extracts from whole testis of *Psma8*^*+/+*^ (WT) and *Psma8*^*-/-*^ (KO) (n = 2 mice). Bar in panels, 10 μm. Welch´s *t*-test analysis: * p<0.05; ** p<0.001; *** p<0.0001.

To investigate the molecular basis of PSMA8 localization in the SC, and considering the alteration of SYCP3 and SYCP1 in *Psma8*^-/-^ spermatocytes (Fig 3F and Fig 5B), we used a candidate gene approach to identify additional putative interactors of PSMA8. We co-transfected *Psma8* with cDNAs encoding each of the known central element proteins (SIX6OS1, SYCE1, SYCE2, SYCE3, and TEX12), and the AE protein SYCP3. As positive controls, we exploited the well-known interaction between SYCE2 and TEX12 [51] (S14C Fig). Surprisingly, we detected specific co-immunoprecipitation of PSMA8 with SIX6OS1 and SYCE3 (Fig 8A and S14B Fig). We were unable to immunoprecipitate transfected SYCP3 (using several tags or antibodies against SYCP3), likely due to the highly complex structures of transfected SYCP3, which prevented to perform co-immunoprecipitation experiments. Because SYCP3 forms filamentous structures in the cytoplasm of transfected cells, termed polycomplexes [52], co-expression of an interacting protein with SYCP3 may lead to its recruitment to polycomplexes [24], an indication of protein interaction. Indeed, we obtained self assembled higher structures when *Psma8* was co-transfected with *Sycp3* (Fig 8B). This SYCP3-dependent cytological interaction was not observed when *Psma7* was co-transfected (Fig 8B), further validating the specificity of the interaction given the extensive protein similarity between both PSMA8 and PSMA7 (92%). To validate this interaction *in vivo*, we performed a detailed analysis of SYCP3 in mouse mutant squashed spermatocytes, a procedure in which no solubilization or protein extraction is performed. We observed SYCP3 aggregates/polycomplexes in the *Psma8*-deficient spermatocytes during prophase I and metaphase I / II (Fig 8C and 8D and S7 Table). SYCP3 accumulated in metaphase II chromosomes as abnormal SYCP3 labeling at the centromeres between sister kinetochores and as aggregates in the cytosol (Fig 3F and Fig 8D). Global accumulation of SYCP3 was also observed by western blot of whole testis under high denaturing conditions (Fig 8E) [53]. Interestingly, it has been previously shown that cultured spermatocytes chemically treated with the proteasome inhibitor MG132 form SYCP3 aggregates [17]. Overall, our results suggest that SYCP3 is targeted for degradation by the PSMA8-containing proteasome and that in the absence of PSMA8 its accumulation could mediate, at least in part, the arrest and apoptosis of spermatocytes.

## Discussion

The testis-specific proteasome is one of the three tissue-specific proteasomes identified in mammals (together with the immunoproteasome and the thymoproteasome); however, little is known about its biochemical and physiological function. The groundbreaking work of Xiao-Bo Qiu and colleagues showing the acetyl-histone preference of the PA200 subunit of the proteasome [5] has provided novel insights into the proteasome-dependent degradation of non-ubiquitylated proteins and led to the designation of spermatoproteasome to the PA200-containing proteasome. However, following the criteria employed for the designation of the thymoproteasome, which were devised based on the restricted expression of its β5t subunit in the thymus [9] (GTEx portal), we suggest that the term spermatoproteasome be restricted exclusively to the PSMA8-containing proteasome instead of the widely expressed PA200 subunit [5].

We have shown that genetic depletion of *Psma8* causes the delocalization and the drastic decrease (loss of detection) of the proteasome activator PA200 in spermatocytes. Accordingly, *Psma8*-deficient spermatocytes accumulate acetylated histones. PSMA8 deficiency is comparatively more severe than that of the PA200 single mutant (subfertile) and of the PA200 and PA28γ double mutant, which do not show an arrest in spermatogenesis despite being infertile *in vivo* but not *in vitro* (spermatozoa are not motile but can fertilize *in vitro* [54]). From a genetic analysis perspective, this result would suggest that PSMA8 has additional functions that are independent of the activators PA200 and PA28γ. Our proteomic analysis, together with other data [10], supports this notion and indicates that PSMA8-containing proteasomes can be associated with other regulators such as the 19S subunit, expanding its targets.

Beyond its role in initiation of histone replacement [34], H4K16ac is involved in the three waves of H2AX phosphorylation during prophase I [55]. We have shown that *Psma8* deficiency causes the accumulation of H4ac and H4K16ac during prophase I. However, we did not observe defects in this process in the form of a different staining pattern for γ-H2AX (leptonema and zygonema), including the expansion of γ-H2AX staining to the chromatin of the sex body (in pachynema). However, the observed premature accumulation of H4K16ac at early round spermatid might cause a defect in histone removal later on in spermiogenesis if the *Psma8*^*-/-*^ mutants spermatids would not have entered apoptosis before this event.

We have shown that spermatoproteasome deficiency causes severe defects in protein turnover of key meiotic players that affect metaphase I/II exit, but not the complex process of meiotic recombination that occurs during prophase I (CO). By using a candidate approach of PIPs, we have identified CDK1 and TRIP13 as likely crucial proteins that have an abnormal expression pattern during meiotic metaphase in mutant mice. Given the key roles of these proteins in all aspects of mitotic/meiotic division (including SAC activation), the accumulation of aberrant metaphase I/II spermatocytes in *Psma8-*deficient mice is to be expected.

The role of CDK1 in the metaphase-anaphase transition is complex and is multifaceted. CDK1 inhibits and activates APC/C by promoting the SAC and also by a SAC-independent mechanism [56]. The balance between these opposing functions determines cyclin B1 destruction and separase activation, giving rise to cohesin cleavage and anaphase onset [57]. Based on the normal expression levels of PTTG1 in *Psma8*^*-/-*^ metaphase I cells, it can be argued that there is no precocious APC activation in *Psma8*-deficient cells (S17 Fig). Given that CDK1 activation of the SAC is dominant over the activation of APC^Cdc20^ [58] in oocytes, we suggest that the former effect is acting on *Psma8-*deficient spermatocytes. The question how CDK1 promotes the SAC is still unresolved in oocytes and even less is known about this in spermatocytes

Another group of proteins found to be deregulated in spermatoproteasome-deficient mice are the SC structural proteins SYCP1 and SYCP3. The precise effect of the accumulated SYCP1 in the cytoplasm of *Psma8*^*-/-*^ spermatocytes cannot be experimentally analyzed. However, the coiled-coil structure and self-assemblance abilities of SYCP1 strongly suggest a functionally detrimental consequence. Similarly, the presence of SYCP3 aggregates during pachynema and metaphase I mutant spermatocytes and its persistence at metaphase II centromeres, where SYCP3 is barely visible in WT cells, also suggest a detrimental effect on these cells causing their entrance into apoptosis.

We have also shown that PSMA8 is delocalized in the severe synapsis *Six6os1* mutant, which is consistent with the observed co-immunoprecipitation of PSMA8 with SYCP1, SIX6OS1 and SYCE3. All the synapsis-less mutants of CE proteins failed to load properly or lacked SYCP1 and the remaining CE proteins [24, 59–61]. Thus, we would predict delocalization of the spermatoproteasome from the SC in the remaining mouse mutants of the CE proteins. Overall, our results support the idea of a physical anchorage or recruitment of the spermatoproteasome to the SC especially through SYCP3, possibly facilitated or mediated by SYCP1, SIX6OS1 and SYCE3 as their most relevant structural partners. Supporting this notion, the Zip1 transverse filament protein of the yeast SC participates in the recruitment of the proteasome to the SC [22], suggesting an evolutionary conservation of the mechanism.

Yeast mutated for a nonessential subunit of the proteasome (pre9) showed abnormal meiotic recombination, pairing and synapsis [22]. Similar but milder defects were also observed in spermatocytes cultured with a proteasome inhibitor [17]. It has been proposed that the UPS regulates the proteostatic turnover of the ZMM which is required for efficient synapsis and CO [17], through the RNF212 (E3 sumo ligase)-Hei10 (E3 ubiquitin ligase) pathway [31]. Given this, the lack of a meiotic recombination phenotype (DSBs are generated and repaired and COs are generated normally) in our *Psma8*-deficient mouse is surprising. It can be argued that PSMA7-containing proteasomes are still present and at the early stages of meiosis are compensating for the loss of function of *Psma8*. Another possible but not mutually exclusive explanation is that the main targets of the PSMA8-containing proteasome are proteins from mid-prophase I onwards.

The spermatoproteasome through its complex interactome would serve as a hub for the fine tuning of several fundamental key molecules of the spermatogenic process such as those analyzed during the present work (SYCP1, SYCP3, TRIP13, CDK1 and acetyl-histones). Our data suggest that deregulation of proteostasis of key meiotic proteins promoting cell division leads to the presence of multipolar spindles and aberrant meiotic exit. Thus, we favor an explanation in which the joint contribution of several pathways is responsible for the observed infertility.

In relation to human disease, protein degradation was one of the top cellular functions found in an unbiased differential proteomic profiling of spermatozoa proteins from infertile men with a varicocele [62]. More specifically, PSMA8 is among the top 7 in this list of proteins that are differentially expressed, suggesting a causal role in the severity of the disease. From an organismal perspective, *Psma8* transcription is mainly restricted to the human testis and to some tumors like Burkit lymphoma and melanoma (TCGC database). Altogether, and considering the PSMA8 dependency of the mouse male germline, we suggest that the spermatoproteasome may be an effective target for male contraception and for the treatment of some human malignancies.

## Material and methods

### *In vivo* electroporation of testes

Testes were freed from the abdominal cavity and 10 μl of DNA solution (50 μg) mixed with 1μl of 10×FastGreen (Sigma Aldrich F7258) was injected into the rete testis with a DNA embryo microinjection tip. After a period of 1 h following the injection, testes were held between electrodes and four electric pulses were applied (35 V for 50 ms each pulse) using a CUY21 BEX electroporator.

### Production of CRISPR/Cas9-Edited mice

Psma8-sgRNAs G71 5’- GGGCATACT CCACTTGGAAA -3’ G84 5’-ACCGCGGTAAGCTGCTCCCC-3’ targeting exon 1 and intron 1 were predicted at crispr.mit.edu. Psma8-sgRNAs were produced by cloning annealed complementary oligos at the BbsI site of pX330 (#42230, Addgene), generating PCR products containing a T7 promoter sequence that were purified (NZYtech), and then *in vitro* transcribed with the MEGAshortscrip T7 Transcription Kit (Life Technologies). The plasmid pST1374-NLS-flag-linker-Cas9 (#44758; Addgene) was used for generating Cas9 mRNA. After linearization with AgeI, it was transcribed and capped with the mMESSAGE mMACHINE T7 Transcription Kit (AM1345; Life Technologies). RNAs were purified using the RNeasy Mini Kit (Qiagen). RNAs (100 ng/μl Cas9 and 50ng/μl each guide RNA) were microinjected into B6/CBA F2 zygotes (hybrids between strains C57BL/6J and CBA/J) [63] at the Transgenic Facility of the University of Salamanca. Edited founders were identified by PCR amplification (Taq polymerase, NZYtech) with primers flanking exons 1 and intron 1 (Primer F 5`-CTTCTCGGTATGACAGGGCAATC-3’ and R 5’- ACTCTACCTCCACTGCCAAC CTG-3’) and either direct sequenced or subcloned into pBlueScript (Stratagene) followed by Sanger sequencing. The predicted best null mutation was selected by PCR sequencing of the targeted region of *Psma8* (S3B Fig). The selected mutant allele was 166 bp long versus 222bp of the wild-type. The founder was crossed with wild-type C57BL/6J to eliminate possible unwanted off-targets. *Psma8*^*+/-*^ heterozygous mice were re-sequenced and crossed to give rise to *Psma8*^*-/-*^ homozygous. Genotyping was performed by analysis of the PCR products of genomic DNA with primers F and R. Mouse mutants for Rec8 and Six6os1 have been previously developed [24, 25].

### Histology

For histological analysis of adult testes, mice were perfused and their testes were processed into serial paraffin sections and stained with hematoxylin-eosin or were fixed in Bouin´s fixative and stained with Periodic acid–Schiff (PAS) and hematoxylin.

### Microscopy

Slides were visualized at room temperature using a microscope (Axioplan 2; Carl Zeiss, Inc.) with 63 × objectives with an aperture of 1.4 (Carl Zeiss, Inc.). Images were taken with a digital camera (ORCA-ER; Hamamatsu) and processed with OPENLAB 4.0.3 and Photoshop (Adobe). Quantification of fluorescence signals was performed using Image J software. Squashed preparations were visualized with a Delta vision microscopy station. Stimulated emission depletion (STED) microscopy (SP8, Leica) was used to generate the super-resolution images. Secondary antibodies for STED imaging were conjugated to Alexa 555 and 488 (Invitrogen). Slides were mounted in Prolong Antifade Gold without DAPI.

### Immunocytology

Testes were detunicated and processed for spreading using a conventional "dry-down" technique or squashing [64]. Antibody against the C-term of PSMA8 was a gift from Dr. Murata (Univ of Tokyo, Japan) and has been previously described [10]. Rabbit polyclonal antibodies against PSMA8 were developed by Proteintech (R1 and R2) against a fusion protein of poly-His with full length PSMA8 (pET vector) of mouse origin (see S1 Fig for validation) and was used to validate the immunofluorescence and western results. The primary antibodies used for immunofluorescence were rabbit αSYCP1 IgG ab15090 (1:200) (Abcam), rabbit anti-γH2AX (ser139) IgG #07–164 (1:200) (Millipore), ACA or purified human α-centromere proteins IgG 15–235 (1:5, Antibodies Incorporated), mouse αMLH1 51-1327GR (1:5, BD Biosciences), mouse αSYCP3 IgG sc-74569 (1:100), rabbit αRAD51 PC130 (1:50, Calbiochem), Mouse αCDK1 sc-54 (1:20 IF; 1:1000 wb, Santa Cruz), rabbit αCDK1 Tyr15p #4539 (1:10, Cell Signaling), rabbit αCDK2 sc-6248 (1:20, Santa Cruz), rabbit αPTTG1 serum K783 (1:20 IF, 1:1000 wb), rabbit αTRIP13 19602-1-AP (1:20, Proteintech), rabbit αH2AL2 (1:100, from Dr. Saadi Khochbin), rabbit αPA200 (1:20, Bethyl A303-880A), rabbit α-Caspase3 #9661 (1:30, Cell Signaling), rabbit αH2AK5ac ab45152 (1:20, Abcam), Rabbit αH4K16ac #07–329 (1:50 Millipore), Rabbit αH3ac (K9 and K14) #06–599 (1:20, Millipore), Rabbit αH4ac (K5, K8, K12 and K16) #06–598 (1:20, Millipore), Mouse αUbiquitin 11023 (1:20 IF, 1:1000 wb, QED Bioscience), Rabbit αHORMAD1 and αHORMAD2 and chicken anti SYCP1 (1:50, from Dr. Attila Toth; [65]), Rabbit anti p-ser10-H3 06–570 (1:100, Millipore), Mouse anti α-tubulin T9026 (1:100, Sigma), Rabbit αCyclin B1 ab72 (1:20, Abcam), Rabbit αMAD2 (1:30 provided by Dr. Stemmann), Peanut agglutinin lectin L7381 (15μg/ml, Sigma), SMC6 ab18039 (1:50, Abcam), Human αVASA 560189 (1:100, BD), Rabbit αINCENP 1186 (1:50, provided by Dr. Earnshaw). TUNEL staining of chromosome spreads was performed with the *in situ* cell death detection kit (Roche).

### FACs analysis

*Psma8*^*+/+*^ and *Psma8*^*−/−*^ testicular cells preparation and measurement of their DNA content were performed by a standard procedure [66]. Briefly, the testes were detunicated and the seminiferous tubules were kept in 5 ml of ice-cold separation medium (DMEM supplemented with 10% FCS, 0.1 mM NEAA, 1.5 mM sodium pyruvate, 4 mM L-glutamine and 75 μg/ml ampicillin). They were treated with 0.1 mg/ml collagenase at 37°C for 10 min under mild shaking. The sedimented seminiferous tubules were washed twice with separation medium and treated for 2 min at 37°C with 2.5 μg/ml trypsin and 1 U/ml DNAse I in separation medium and transferred to ice. Afterwards, single cells were extracted from the seminiferous cords with a Pasteur pipette and filtered through a 40 μm nylon mesh. The cell suspension (2 × 10^6^ cells/ml) was diluted 1:1 with a solution containing 0.05 mg/ml propidium iodide and 0.1 mg/ml RNAse for 15 min. Finally, the cells were analyzed through flow cytometry in a cytometer FACSCalibur and the BD Cell-Quest software. The cell cycle distribution was analyzed with the Kaluza Analysis software (Beckman Coulter).

### Proteasome assay

The 26S proteasome assay was carried out in a total volume of 250 μl in 96 well plates with 2 mM ATP in 26S buffer using 100 μg of protein supernatants from whole extracts of mouse testis. Fluorescently labeled substrates employed were: succinyl-Leu-Leu-Val-Tyr-7-amino-4-methylcoumarin (Suc-LLVY-AMC), Z-Ala-Arg-Arg-AMC (Z-ARR-AMC, Bachem), and Z-Leu-Leu-Glu-AMC (Z-LLE-AMC) for the detection of the chymotrypsin- (β5 catalytic subunit), trypsin- (β2 catalytic subunit) and caspase- (β1 catalytic) like activity measurements respectively. The final substrate concentration in each assay was 100 μM.

### Cell lines

The HEK293T, GC1-spg, Leydig TM3, and Sertoli TM4 cell lines were directly purchased at the ATCC and cultured in standard cell media. HEK293T cell line was transfected with Lipofectamine (Invitrogen) or Jetpei (PolyPlus). Cell lines were tested for mycoplasma contamination (Mycoplasma PCR ELISA, Sigma).

### Generation of plasmids

Full-length cDNAs encoding PSMA8, PSMA7, CDK1, SYCP1 and SIX6OS1, SYCP3, SYCE2, TEX12, TEX30, PIWIL1 and PIWIL2 were RT-PCR amplified from murine testis RNA. Full-length cDNAs were cloned into the EcoRV pcDNA3-2XFlag or SmaI pEGFP-C1 expression vectors under the CMV promoter. In frame cloning was verified by Sanger sequencing.

### Immunoprecipitation and western blotting

200 μg of antibody R1 and R2 were bound to 100 μl of sepharose beads slurry (GE Healthcare). Testis extracts were prepared in 50mM Tris HCl (pH8), 500mM NaCl, 1mM EDTA 1% tritonX-100. 20 mg of proteins extracts were incubated o/n with the Sepharose beads. Protein-bound beads were packed into columns and washed in extracting buffer for three times. Protein were eluted in 100 mM glycine pH3. The whole immunoprecipitation of PSMA8 was performed in a buffer lacking ATP and glycerol to increase the stringency of the interactors and regulators/activators subunits. HEK293T cells were transiently transfected and whole cell extracts were prepared and cleared with protein G Sepharose beads (GE Healthcare) for 1 h. The antibody was added for 2 h and immunocomplexes were isolated by adsorption to protein G-Sepharose beads o/n. After washing, the proteins were eluted from the beads with 2xSDS gel-loading buffer 100mM Tris-Hcl (pH 7), 4% SDS, 0.2% bromophenol blue, 200mM β-mercaptoethanol and 20% glycerol, and loaded onto reducing polyacrylamide SDS gels. The proteins were detected by western blotting with the indicated antibodies. Immunoprecipitations were performed using mouse αFlag IgG (5μg; F1804, Sigma-Aldrich), mouse αGFP IgG (4 μg; CSB-MA000051M0m, Cusabio), rabbit αMyc Tag IgG (4μg; #06–549, Millipore), mouse αHA.11 IgG MMS- (5μL, aprox. 10μg/1mg prot; 101R, Covance), ChromPure mouse IgG (5μg/1mg prot; 015-000-003), ChomPure rabbit IgG (5μg/1mg prot.; 011-000-003, Jackson ImmunoResearch), ChomPure goat IgG (5μg/1mg prot.; 005-000-003, Jackson ImmunoResearch). Primary antibodies used for western blotting were rabbit αFlag IgG (1:2000; F7425 Sigma-Aldrich), goat αGFP IgG (sc-5385, Santa Cruz) (1:3000), rabbit αHA IgG (H6908, Sigma-Aldrich) (1:1.000), mouse αMyc obtained from hybridoma cell myc-1-9E10.2 ATCC (1:5). Secondary horseradish peroxidase-conjugated α-mouse (715-035-150, Jackson ImmunoResearch), α-rabbit (711-035-152, Jackson ImmunoResearch), or α-goat (705-035-147, Jackson ImmunoResearch) antibodies were used at 1:5000 dilution. Antibodies were detected by using Immobilon Western Chemiluminescent HRP Substrate from Millipore. Protein extracts for the analysis of SYCP3, CDK1 and CyclinB1 were extracted in Tris-HCl 250mM, SDS10%, Glycerol 50% (denaturing buffer).

### MS/MS data analysis

Raw MS data were analized using MaxQuant (v. 1.5.7.4) and Perseus (v. 1.5.6.0) programmes 71. Searches were generated versus the *Mus musculus* proteome (UP000000589, May 2017 release) and Maxquant contaminants. All FDRs were of 1%. Variable modifications taken into account were oxidation of M, acetylation of the N-term and ubiquitylation remnants di-Gly and LRGG, while fixed modifications included considered only carbamidomethylation of C. The maximum number of modifications allowed per peptide was 5. For the case of the protein group of CDK1 to 3, experimental results showed that the protein detected was CDK1. For the PSMA8 antibodies R1 and R2, ratios of their respective iBAQ intensity versus the correspondent iBAQ intensity in the control sample were calculated. Proteins with ratio higher or equal to 5 and two or more unique peptides for at least one RP antibody were selected for ulterior analysis. Additionally, in order to avoid filtering rare proteins, those with at least one unique peptide and one peptide for both Rabbit antibodies (R1 and R2) and none for anti-IgG were also selected for further analysis.

### Functional and pathway analysis

GO and KEGG over-representation tests were performed using the R package *clusterProfiler* [67] using standard parameters except for a FDR cutoff of 0.01. KEGG pathways where some key genes (TRIP13, CDK1, SYCP1, DDX4, SYCP3, SYCE3, SIX6OS1) operate and the role of the co-immunoprecipitated proteins were studied using the R package *pathview* [68].

### Statistics

In order to compare counts between genotypes at different stages, we used the Welch´s t-test (unequal variances t-test), which was appropriate as the count data were not highly skewed (i.e., were reasonably approximated by a normal distribution) and in most cases showed unequal variance. We applied a two-sided test in all the cases. Asterisks denote statistical significance: *p-value <0.01, **p-value <0.001 and ***p-value<0.0001.

### Ethics statement

Mice were housed in a temperature-controlled facility (specific pathogen free, spf) using individually ventilated cages, standard diet and a 12 h light/dark cycle, according to EU laws at the “Servicio de Experimentación Animal, SEA”. Mouse protocols were approved by the Ethics Committee for Animal Experimentation of the University of Salamanca (USAL). We made every effort to minimize suffering and to improve animal welfare. Blinded experiments were not possible since the phenotype was obvious between wild type and *Psma8*-deficient mouse for all of the experimental procedures used. No randomization methods were applied since the animals were not divided in groups/treatments. The minimum size used for each analysis was two animals/genotype.

## Supporting information

S1 Fig Validation of the antibodies raised against PSMA8. (A) HEK293T cells were transfected with a plasmid encoding PSMA8-GFP, PSMA7-GFP or GFP and the whole extracts were analyzed by western blot using rabbit α-PSMA8 C-terminal (left panel, α4S), rabbit α-PSMA8 (central panel, R2) and α-GFP (right panel, GFP). Immunodetection of β-actin was used as loading control. The rabbit α-α4S antibody detected exclusively the 60 kDa band representing PSMA8-GFP. The rabbit α-PSMA8 R2 antibody detected both bands representing PSMA8-GFP and PSMA7-GFP. The bands of 60 kDa (PSMA7 and PSMA8) and 30 kDa (GFP) were all detected with the goat α-GFP validating the experiments. (B) Immunofluorescence of HEK293T cells transfected with plasmids encoding PSMA8-GFP, PSMA7-GFP or GFP. Both PSMA8 and PSMA7 were detected with rabbit α-PSMA8-R2 (red) and GFP by direct fluorescence signal (green). Green and red signals co-localize in the cytoplasm of the transfected HEK293T cells. The experiments were reproduced three times. Bar represents 10 μm.
(TIF)

S2 Fig Localization of PSMA8 in mouse spermatocytes. (A) Double immunolabeling of endogenous PSMA8 (R2 antibody, green) and SYCP3 (red) in mouse spermatocytes. From the leptotene to zygotene stage, PSMA8 is detected at the synapsed autosomal LEs. At pachytene, PSMA8 is located at the totally synapsed axes and at the PAR of the sex XY bivalent. In diplotene, PSMA8 localizes at the still synapsed AEs and disappears at diakinesis. (B) Double immunolabeling of spermatocytes spread preparations with PSMA8 (green) and SYCP1 (red), showing that PSMA8 localizes to the synapsed LEs but do not perfectly co-localize with SYCP1 (upper panel). Magnification of the XY bivalent (lower panel) showing the PAR (arrow). Bars represent 10 μm (A and B, upper panel) and 1.5 μm (B, lower panel).
(TIF)

S3 Fig Generation and genetic characterization of *Psma8*-deficient mice. (A) Diagrammatic representation of the mouse *Psma8* locus (WT) and the genome editing strategy showing the sgRNAs located on exon 1 and intron 1 (see methods), the corresponding coding exons (light grey) and non-coding exons (open boxes). Thin (non-coding) and thick (coding sequences) lines under exons represent the expected transcript derived from wild-type (black) and *Psma8* edited allele (blue). ATG, initiation codon; TGA and *, stop codon. The nucleotide sequence of the 56 base pair deletion derived from PCR amplification of DNA from the *Psma8* ^*edited/edited*^ is indicated (Δ). Primers (F and R) are represented by arrows. (B) PCR analysis of genomic DNA from three littermate progeny of *Psma8*^*+/-*^ heterozygote crosses. The PCR amplification with primers F and R revealed 222 and 166 bp fragments for wild-type and disrupted alleles respectively. Wild-type (WT, +/+), heterozygous (Het, +/-), and homozygous knock-out (KO, -/-) animals. (C) Western blot analysis of protein extracts from wild type testis (P22 and adult), KO testis (P16, P22 and adult) with a specific antibody against the C-terminal (α4S) and whole recombinant PSMA8 protein (PSMA8-R2). β-actin was used as loading control. The corresponding bands to PSMA8 and PSMA7 are indicated in the right of the panel. Note that at the P22 and in adult stages the intensity of both bands abolishes its independent observation. (D) Double immunofluorescence of spermatocytes at pachytene stage obtained from *Psma8*^*+/+*^ and *Psma8*^*-/-*^ mice using SYCP3 (red) and PSMA8 (R2 antibody, green). Green labeling in *Psma8*^*-/-*^ spermatocytes (49% of the wild type) represents cross-reactivity of the antiserum with PSMA7. Plot under the image panel represents the quantification of intensity from *Psma8*^*+/+*^ and *Psma8*^*-/-*^ spermatocytes. Welch´s *t*-test analysis: * p<0.01. Bar in panel, 10 μm.
(TIF)

S4 Fig Validation of the identity of round spermatids with molecular markers. (A) PNA staining (green) of acrosome in spread preparations from wild type and *Psma8*^*-/-*^ cells. Double labeling of squash tubules of VASA (chromatoid body), INCENP [1], SMC6 [2] (green) with SYCP3 (red) from wild type and *Psma8*^*-/-*^ mice. The combined labeling of INCENP (labels both interkinesis and round spermatids, [1]) and SYCP3 (mainly labels interkinesis with a typical barr patterning at the chromocenters, see below S4B Fig) is compatible with round spermatids. The combined double immunolabeling of SMC6 (labels both interkinesis and round spermatids, [2]) and SYCP3 (mainly labels interkinesis with a typical barr patterns at the chromocenters, see below S4B Fig) is also compatible being round spermatids. (B) Double labeling of SYCP3 (green) and ACA (red) showing the different pattern of secondary spermatocytes at interkinesis and round spermatids. Bars in panels represent 10 μm (A, PNA panel) and 5 μm (rest of panels).
(TIF)

S5 Fig Early arrest of *Psma8^-/-^* spermatids and gating strategy of the FACs analysis. (A) Immunolabeling of H2AL2 (green) show positive staining in elongating spermatids from wild type mice but lack of staining in *Psma8*^*-/-*^ mice. Chromatin was stained with DAPI. Bar represents 10 μm. (B) Gating strategy employed in the FACs analysis of Fig 3D. Grey dots represent cells that were excluded from the analysis whilst dots included in the polygon represent cells that were employed for the analysis. Red dots enclose 1C cells, blue dots represent 2C cells and green dots enclose 4C cells.
(TIF)

S6 Fig Normal synapsis and desynapsis in spermatocytes lacking PSMA8. Double immunolabeling of SYCP3 (red) and SYCP1 (green) showing normal synapsis and desynapsis from early zygotene to diakinesis in *Psma8*^*-/-*^ in comparison with *Psma8*^*+/+*^. Bar represents 10 μm.
(TIF)

S7 Fig DSBs are generated and repaired as COs in spermatocytes lacking PSMA8. (A) Double immunolabeling of γ-H2AX (green) with SYCP3 (red) in wild-type and *Psma8*^*-/-*^ spermatocytes from leptotene to diplotene (upper panel). In WT and KO leptonemas, γ-H2AX labels intensely the chromatin. After repair, γ-H2AX labeling remains only in the chromatin of the sex body of the pachynemas. Plot right to the panel represent the quantification of the fluorescence intensity from *Psma8*^*+/+*^ and *Psma8*^*-/-*^ spermatocytes at leptotene and pachytene. Late round spermatids (LR) but not early round spermatids (ER) from wild type mice show positive staining for γ-H2AX but these highly differentiated cells are lacking in the *Psma8*^*-/-*^ tubules which are arrested at early round spermatids without γ-H2AX staining (bottom panel). (B) Double immunolabeling of SYCP3 (red) and RAD51 (green). RAD51 foci associates to the AEs in leptonema spermatocytes of both genotypes (similar number of foci) and dissociate towards pachytene with a similar kinetics. Plot right to the image panel represents the quantification of the number of foci from *Psma8*^*+/+*^ and *Psma8*^*-/-*^ spermatocytes. (C) Double immunolabeling of SYCP3 (red) with MLH1 (green). MLH1 foci are present along each autosomal SC in wild-type and *Psma8*^*-/-*^ pachynema meiocytes in a similar way. Plot right to the panel represents the quantification of the values of the MLH1 foci from *Psma8*^*+/+*^ and *Psma8*^*-/-*^ spermatocytes. Bars represent 10 μm. Welch´s *t*-test analysis: * p<0.01; ** p<0.001; *** p<0.0001. Quantification data is indicated in S3 Table.
(TIF)

S8 Fig PA200 localization in prophase I from *Psma8^+/+^* and *Psma8^-/-^* spermatocytes. Double immunolabeling of PA200 (green) and SYCP3 (red) in chromosome spreads from zygotene to diakinesis. PA200 is detected at the chromosome axes in wild type spermatocytes in contrast to the absence of labeling in *Psma8*^*-/-*^ spermatocytes. Bar in panels, 10 μm.
(TIF)

S9 Fig PSMA8 deficiency provokes an slight increase of H2AK5ac at prophase I. Double immunolabeling of H2AK5ac (green) with SYCP3 (red) in wild-type (left panel) and *Psma8*^*-/-*^ spermatocytes (right panel). In WT and KO spermatocytes chromatin start to be labelled at early pachytene around chromosomes axes. Plots from each panel representing the quantification of fluorescence intensity from *Psma8*^*+/+*^ and *Psma8*^*-/-*^ spermatocytes are depicted in Fig 4A. Bar represents 10 μm.
(TIF)

S10 Fig PSMA8 deficiency provokes an slight increase of H3ac at prophase I. Double immunolabeling of H3ac (green) with SYCP3 (red) in wild-type (left panel) and *Psma8*^*-/-*^ spermatocytes (right panel). Spermatocytes from *Psma8*^*+/+*^ and *Psma8*^*-/-*^ show labeling for H3ac at early pachytene in a very diffuse manner surrounding chromosomes axes. Plots from each panel representing the quantification of fluorescence intensity from *Psma8*^*+/+*^ and *Psma8*^*-/-*^ spermatocytes are in Fig 4B. Bar represents 10 μm.
(TIF)

S11 Fig PSMA8 deficiency provokes an slight increase of H4ac at prophase I and in round spermatids. Double immunolabeling of H4ac (green) with SYCP3 (red) in wild-type and *Psma8*^*-/-*^ spermatocytes. Spermatocytes from *Psma8*^*+/+*^ and *Psma8*^*-/-*^ show labeling for H4ac in a very diffuse manner surrounding chromosomes from pachytene to metaphase I (right panel). In wild type metaphase I, H4ac labeling appears weakly painting the chromosomes and on some of the centromeres. However, *Psma8*-deficient cells show a more intense labeling specially at the centromeres (lower panel). Round spermatid from *Psma8*^*-/-*^ accumulates H4ac labeling at the chromatin in comparison with the WT. Plots from each panel representing the quantification of fluorescence intensity from *Psma8*^*+/+*^ and *Psma8*^*-/-*^ spermatocytes are in Fig 4C. Bars represent 10 μm.
(TIF)

S12 Fig PSMA8 deficiency provokes an increase of H4K16ac at prophase I and in metaphase I / round spermatids. Double immunolabeling of H4K16ac (green) with SYCP3 (red) in wild-type and *Psma8*^*-/-*^ spermatocytes. Spermatocytes from *Psma8*^*+/+*^ and *Psma8*^*-/-*^ show labeling for H4K16ac in a very diffuse manner surrounding chromosomes from pachytene to metaphase I (right panel). In wild type metaphase I, H4K16ac labeling appears weakly painting the chromosomes. However, *Psma8*-deficient cells show enhance labeling in the chromosomes of metaphase I cells (lower panel). Round spermatid from *Psma8*^*-/-*^ accumulates H4K16ac labeling at the chromatin in comparison with the WT. Plots from each panel representing the quantification of fluorescence intensity from *Psma8*^*+/+*^ and *Psma8*^*-/-*^ spermatocytes are in Fig 4D. Bars represent 10 μm.
(TIF)

S13 Fig PSMA8 deficiency alters Ubiquitylation of mouse spermatocytes. (A) Double immunolabeling of Ubiquitin (green) and SYCP3 (red) in mouse chromosome spreads at pachytene stage from *Psma8*^*+/+*^ and *Psma8*^*-/-*^mice. (B) Double immunolabeling of Ubiquitin (green) and SYCP3 (red) in mouse squashed tubules from *Psma8*^*+/+*^ and *Psma8*^*-/-*^ mice. Chromatin was stained with DAPI. Bars represent 10 μm (A) and 5 μm (B).
(TIF)

S14 Fig Lack of co-immunoprecipitation of PSMA8 with candidate interactors. (A-B) HEK293T cells were co-transfected with GFP-TEX30, GFP-PIWIL1, GFP-PIWIL2, GFP-SYCE1, GFP-SYCE2, and GFP-TEX12, and with Flag-PSMA8. PSMA8 does not co-immunoprecipitates (co-IP) with any of them. (C) Positive control was generated by transfecting HEK293T cells with Flag-SYCE2 and GFP-TEX12. Protein complexes were immunoprecipitated overnight with either an anti-Flag or anti-EGFP or IgGs (negative control) and were analyzed by immunoblotting with the indicated antibody.
(TIF)

S15 Fig CDK1 / Cyclin B1, but not CDK2, are accumulated in *Psma8* mutant spermatocytes. (A) Double immunolabeling of endogenous CDK2 (green) and SYCP3 (red) in WT and KO mouse chromosome spreads at pachytene and metaphase I showing similar labeling at the telomeres and centromeres, respectively. (B) Double immunolabeling of CDK1 (green) and SYCP3 (red) in mouse squashed metaphases I from *Psma8*^*+/+*^ and *Psma8*^*-/-*^mice showing CDK1 accumulation. Plot right to the panel represents the quantification of total CDK1 fluorescence intensity from *Psma8*^*+/+*^ and *Psma8*^*-/-*^ metaphase I cells. (C) Double immunolabeling of Cyclin B1 (green) and SYCP3 (red) in mouse squashed tubules from *Psma8*^*+/+*^ and *Psma8*^*-/-*^ mice showing CyclinB1 accumulation. Plot right to the panel represents the quantification of total CyclinB1 fluorescence intensity in metaphase I cells. Bars represent 10 μm (A), and 5 μm (B,C). Welch´s *t*-test analysis: * p<0.01; ** p<0.001; *** p<0.0001.
(TIF)

S16 Fig HORMADs are not affected by the increased expression of TRIP13 in the *Psma8^-/-^* spermatocytes. (A-B) Double immunolabeling of HORMAD1 (A) and HORMAD2 (B) (green) with SYCP3 (red) in *Psma8*^*+/+*^ and *Psma8*^*-/-*^ spermatocytes at zygotene and pachytene stages. As synapsis progresses HORMAD1 and HORMAD2 are released from the AEs and maintained at the AE of the sex body similarly in the wild type and in the mutant spermatocytes. Bars represent 10 μm.
(TIF)

S17 Fig PTTG1 expression is not altered in the absence of PSMA8. Double immunofluorescence of PTTG1 (green) and SYCP3 (red) in metaphase I cells showing similar expression levels of PTTG1. Plot under the panel represents the quantification of the fluorescence intensity from *Psma8*^*+/+*^ and *Psma8*^*-/-*^ metaphase I cells. Bar in panels, 10 μm. Welch´s *t*-test analysis: * p<0.01; ** p<0.001; *** p<0.0001.
(TIF)

S1 Table Fertility assessment of *Psma8^+/+^*, *Psma8^+/-^* and *Psma8^-/-^* mice. (PDF)

S2 Table Quantification of metaphases I/II in Psma8-/- testis. (A) Quantification of the proportion of tubules with metaphase I/II in PAS stained tubule sections from the histology example shown in Fig 2B. (B) Quantification of the number of metaphase I and II cells present in p-Ser10-H3 stained tubules that show meiotic divisions (Fig 2C). (C) Quantification of the percentage of metaphases-anaphases I and metaphases-anaphases II in squash preparations (double immunolabeled with ACA and SYCP3) measured as the N° of Metaphase-Anaphase I/II divided by the N° of cells (prophase I + Metaphase-Anaphase I + Interkinesis +Metaphase-Anaphase II) (Fig 2D). Apoptotic Metaphase-Anaphase I and Metaphase-Anaphase II within each genotype are indicated.
(PDF)

S3 Table Quantification of γH2AX levels, RAD51 foci, and MLH1 foci (S7 Fig). (PDF)

S4 Table Proteasome subunits and proteasome regulators co-immunoprecipitated with PSMA8 from *Psma8^+/+^*and *Psma8^-/-^* testis protein extracts using anti-PSMA8 R2 antibody. (PDF)

S5 Table Proteasome subunits and proteasome regulators co-immunoprecipitated with PSMA8 selected after analysis and filtering of the data. (PDF)

S6 Table Selection of some of the proteasome-related proteins co-immunoprecipitated with PSMA8 selected after analysis and filtering of the data. (PDF)

S7 Table Quantification of the percentage of spermatocytes showing SYCP3 aggregates during prophase I stages in squash of seminiferous tubules of *Psma8^+/+^* and *Psma8^-/-^* testis. They have been classified in cells with small or large aggregates (n = 2 mice).
(PDF)

S1 Text Exploratory representation of representative KEGG pathways. (A) Cell cycle (mmu04110). (B) Progesterone-mediated oocyte maduration (mmu04914). (C) Oocyte meiosis (mmu04114). In red, proteins detected in the co-IP experiment over the established cut-off.
(HTM)

S2 Text Supporting information references. (DOCX)
