# Supplementary figures and images for: The PSMA8 subunit of the spermatoproteasome is essential for proper meiotic exit and mouse fertility
Source: PLoS Genet. 2019 Aug 22;15(8):e1008316. doi: 10.1371/journal.pgen.1008316 (PMC6726247; doi:10.1371/journal.pgen.1008316)

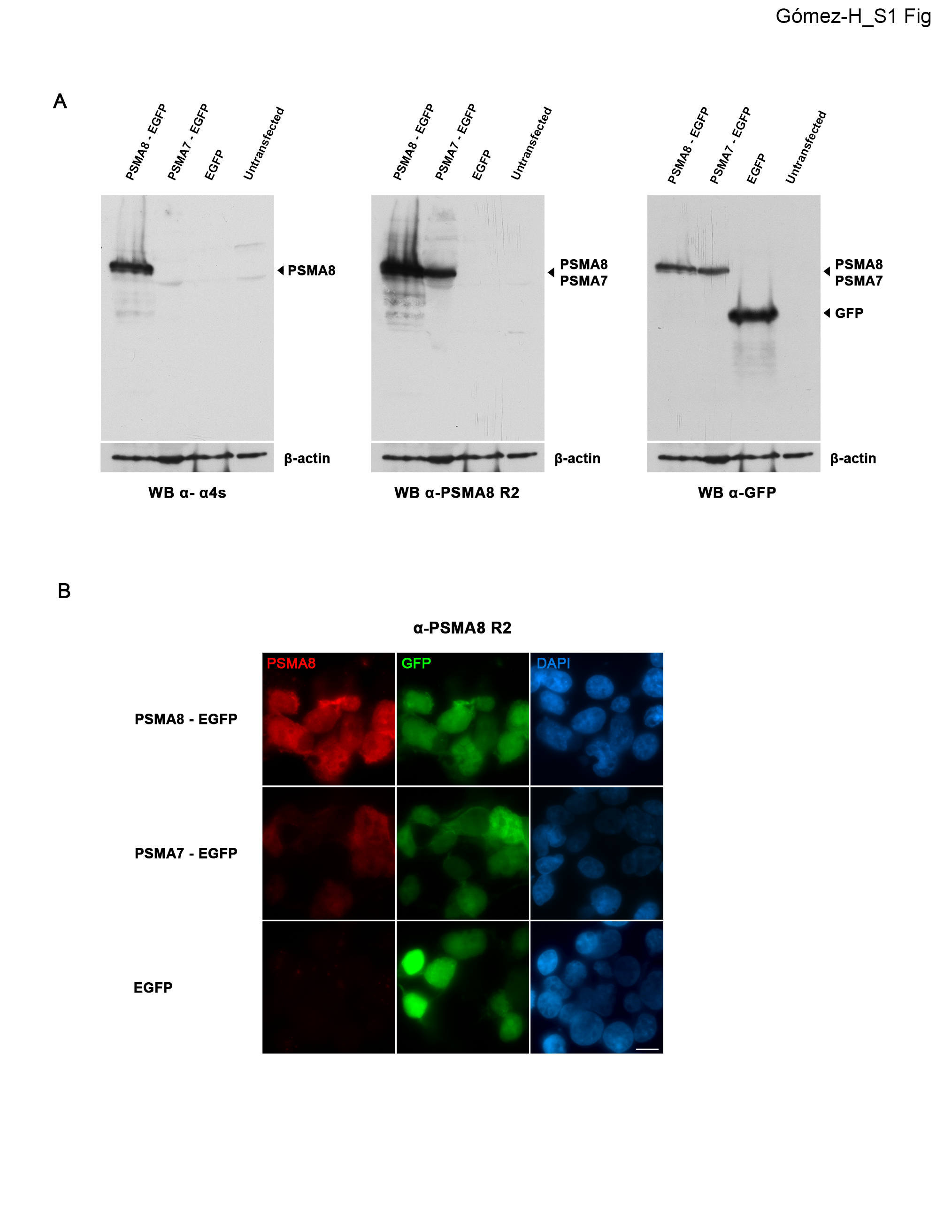

Supplement: S1 Fig — (A) HEK293T cells were transfected with a plasmid encoding PSMA8-GFP, PSMA7-GFP or GFP and the whole extracts were analyzed by western blot using rabbit α-PSMA8 C-terminal (left panel, α4S), rabbit α-PSMA8 (central panel, R2) and α-GFP (right panel, GFP). Immunodetection of β-actin was used as loading control. The rabbit α-α4S antibody detected exclusively the 60 kDa band representing PSMA8-GFP. The rabbit α-PSMA8 R2 antibody detected both bands representing PSMA8-GFP and PSMA7-GFP. The bands of 60 kDa (PSMA7 and PSMA8) and 30 kDa (GFP) were all detected with the goat α-GFP validating the experiments. (B) Immunofluorescence of HEK293T cells transfected with plasmids encoding PSMA8-GFP, PSMA7-GFP or GFP. Both PSMA8 and PSMA7 were detected with rabbit α-PSMA8-R2 (red) and GFP by direct fluorescence signal (green). Green and red signals co-localize in the cytoplasm of the transfected HEK293T cells. The experiments were reproduced three times. Bar represents 10 μm. (TIF) [file pgen.1008316.s001.tif]

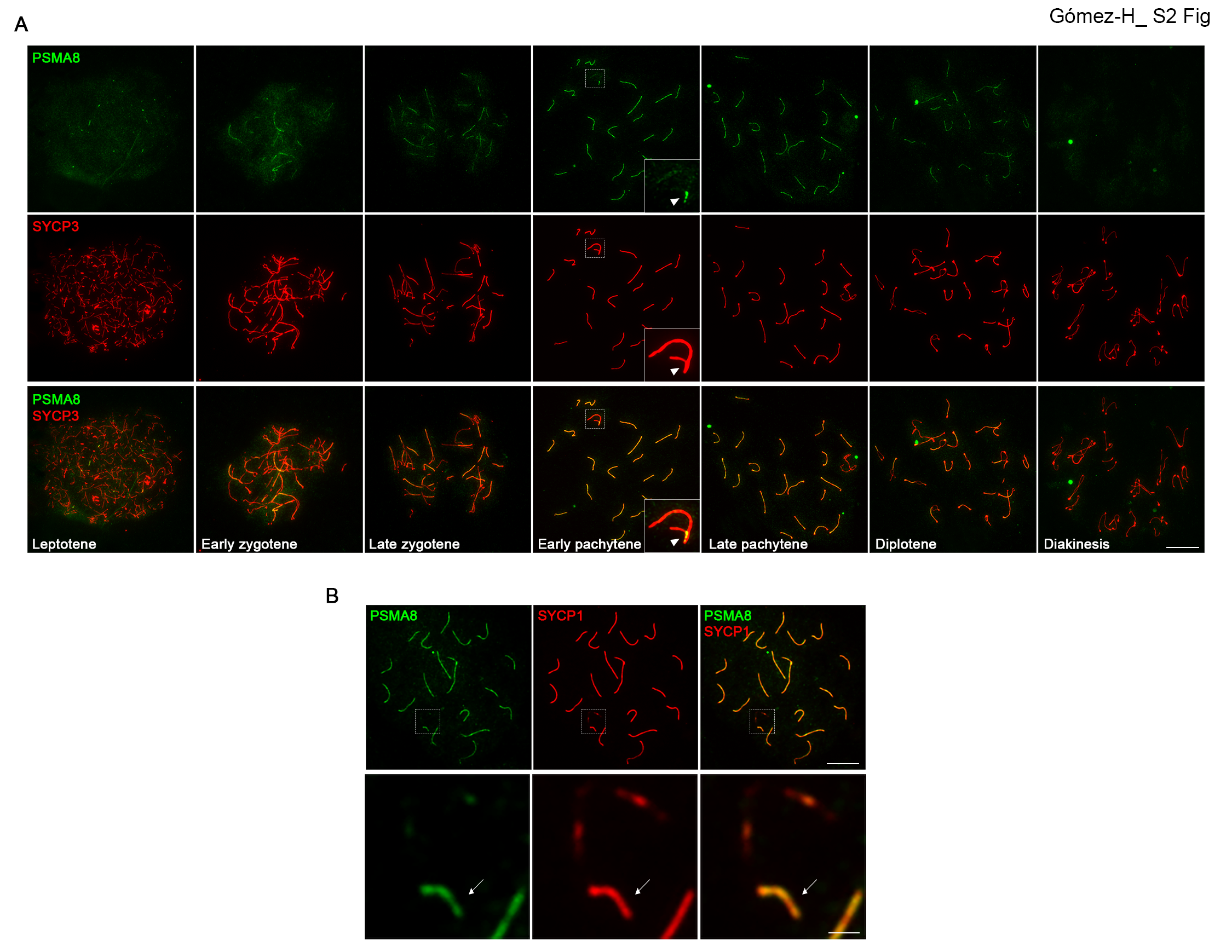

Supplement: S2 Fig — (A) Double immunolabeling of endogenous PSMA8 (R2 antibody, green) and SYCP3 (red) in mouse spermatocytes. From the leptotene to zygotene stage, PSMA8 is detected at the synapsed autosomal LEs. At pachytene, PSMA8 is located at the totally synapsed axes and at the PAR of the sex XY bivalent. In diplotene, PSMA8 localizes at the still synapsed AEs and disappears at diakinesis. (B) Double immunolabeling of spermatocytes spread preparations with PSMA8 (green) and SYCP1 (red), showing that PSMA8 localizes to the synapsed LEs but do not perfectly co-localize with SYCP1 (upper panel). Magnification of the XY bivalent (lower panel) showing the PAR (arrow). Bars represent 10 μm (A and B, upper panel) and 1.5 μm (B, lower panel). (TIF) [file pgen.1008316.s002.tif]

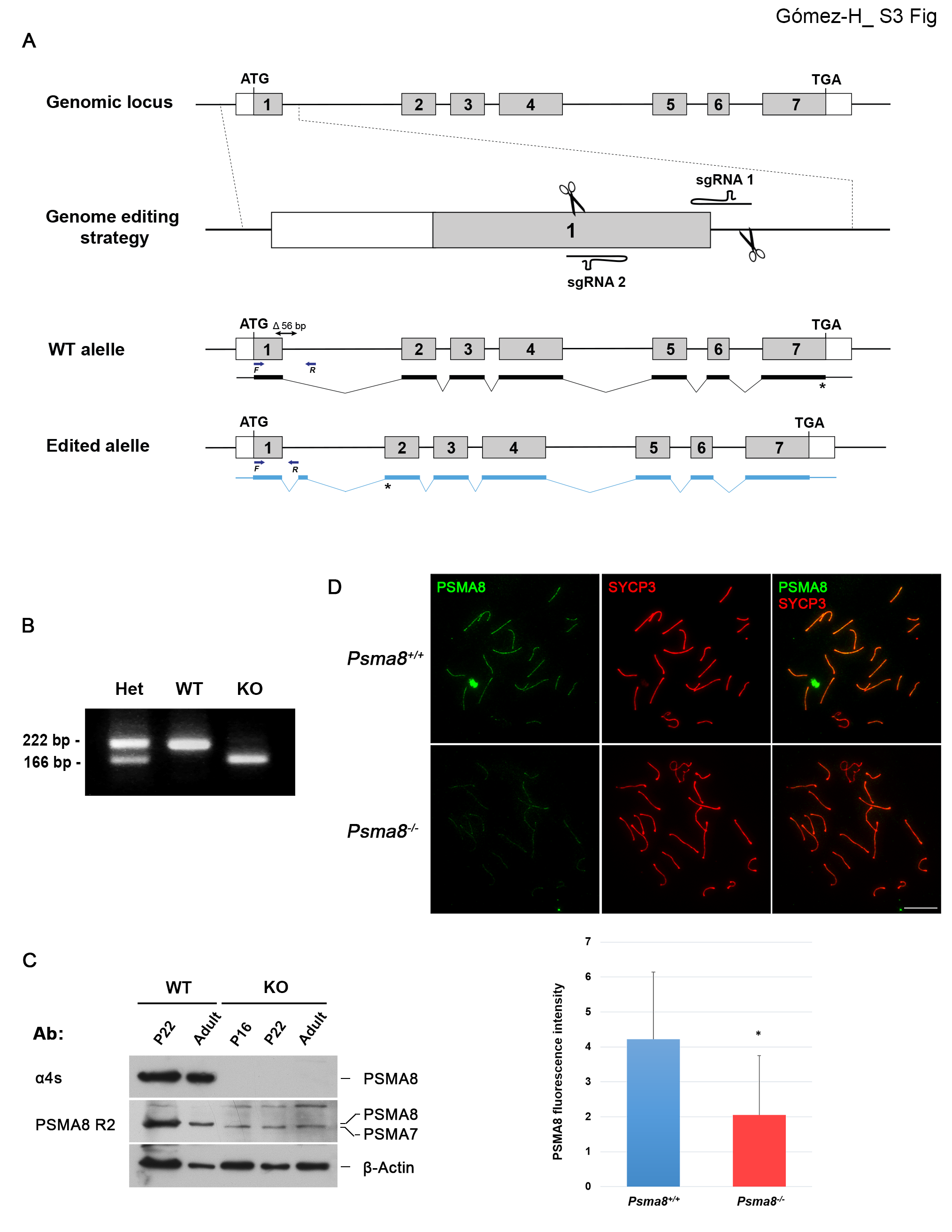

Supplement: S3 Fig — (A) Diagrammatic representation of the mouse Psma8 locus (WT) and the genome editing strategy showing the sgRNAs located on exon 1 and intron 1 (see methods), the corresponding coding exons (light grey) and non-coding exons (open boxes). Thin (non-coding) and thick (coding sequences) lines under exons represent the expected transcript derived from wild-type (black) and Psma8 edited allele (blue). ATG, initiation codon; TGA and *, stop codon. The nucleotide sequence of the 56 base pair deletion derived from PCR amplification of DNA from the Psma8 edited/edited is indicated (Δ). Primers (F and R) are represented by arrows. (B) PCR analysis of genomic DNA from three littermate progeny of Psma8+/- heterozygote crosses. The PCR amplification with primers F and R revealed 222 and 166 bp fragments for wild-type and disrupted alleles respectively. Wild-type (WT, +/+), heterozygous (Het, +/-), and homozygous knock-out (KO, -/-) animals. (C) Western blot analysis of protein extracts from wild type testis (P22 and adult), KO testis (P16, P22 and adult) with a specific antibody against the C-terminal (α4S) and whole recombinant PSMA8 protein (PSMA8-R2). β-actin was used as loading control. The corresponding bands to PSMA8 and PSMA7 are indicated in the right of the panel. Note that at the P22 and in adult stages the intensity of both bands abolishes its independent observation. (D) Double immunofluorescence of spermatocytes at pachytene stage obtained from Psma8+/+ and Psma8-/- mice using SYCP3 (red) and PSMA8 (R2 antibody, green). Green labeling in Psma8-/- spermatocytes (49% of the wild type) represents cross-reactivity of the antiserum with PSMA7. Plot under the image panel represents the quantification of intensity from Psma8+/+ and Psma8-/- spermatocytes. Welch´s t-test analysis: * p<0.01. Bar in panel, 10 μm. (TIF) [file pgen.1008316.s003.tif]

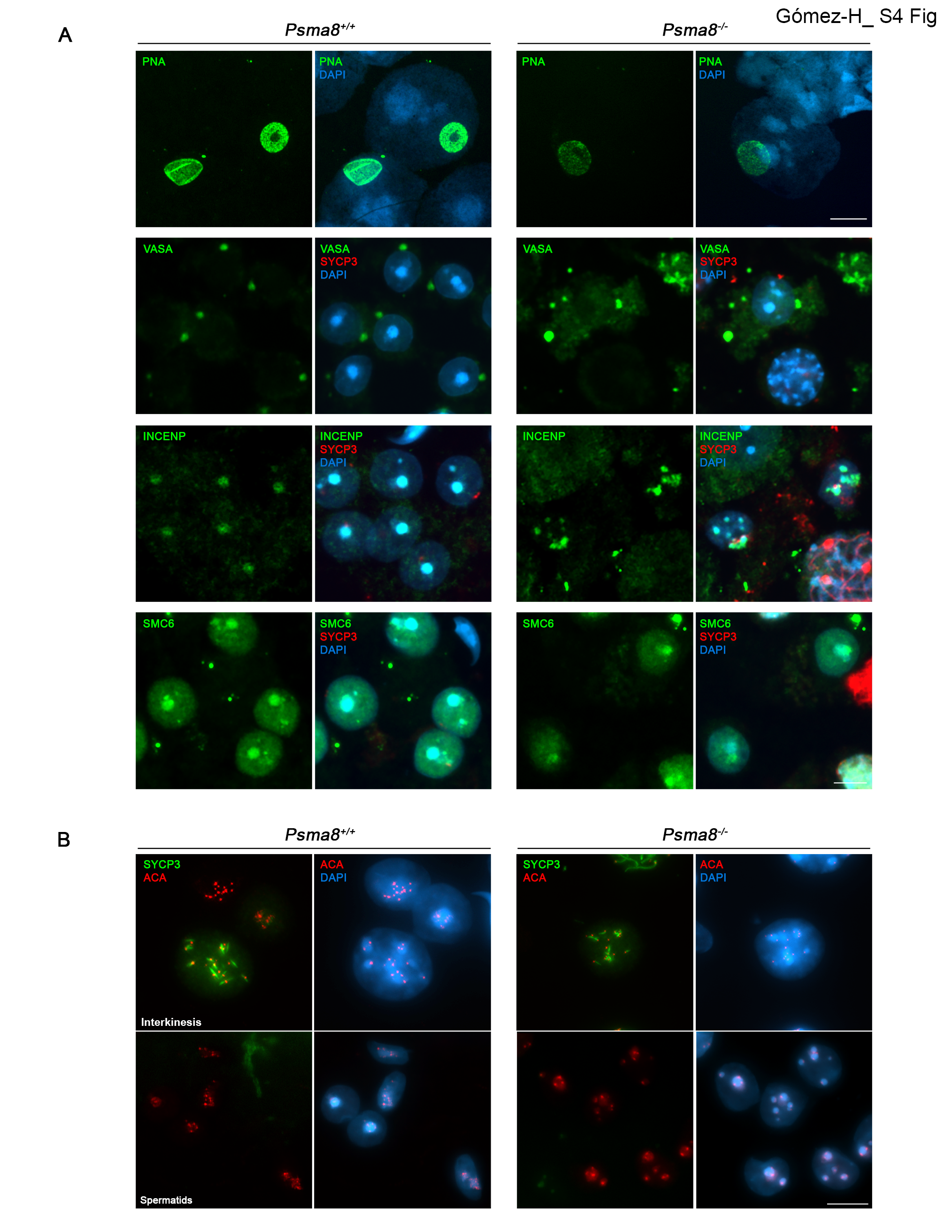

Supplement: S4 Fig — (A) PNA staining (green) of acrosome in spread preparations from wild type and Psma8-/- cells. Double labeling of squash tubules of VASA (chromatoid body), INCENP [1], SMC6 [2] (green) with SYCP3 (red) from wild type and Psma8-/- mice. The combined labeling of INCENP (labels both interkinesis and round spermatids, [1]) and SYCP3 (mainly labels interkinesis with a typical barr patterning at the chromocenters, see below S4B Fig) is compatible with round spermatids. The combined double immunolabeling of SMC6 (labels both interkinesis and round spermatids, [2]) and SYCP3 (mainly labels interkinesis with a typical barr patterns at the chromocenters, see below S4B Fig) is also compatible being round spermatids. (B) Double labeling of SYCP3 (green) and ACA (red) showing the different pattern of secondary spermatocytes at interkinesis and round spermatids. Bars in panels represent 10 μm (A, PNA panel) and 5 μm (rest of panels). (TIF) [file pgen.1008316.s004.tif]

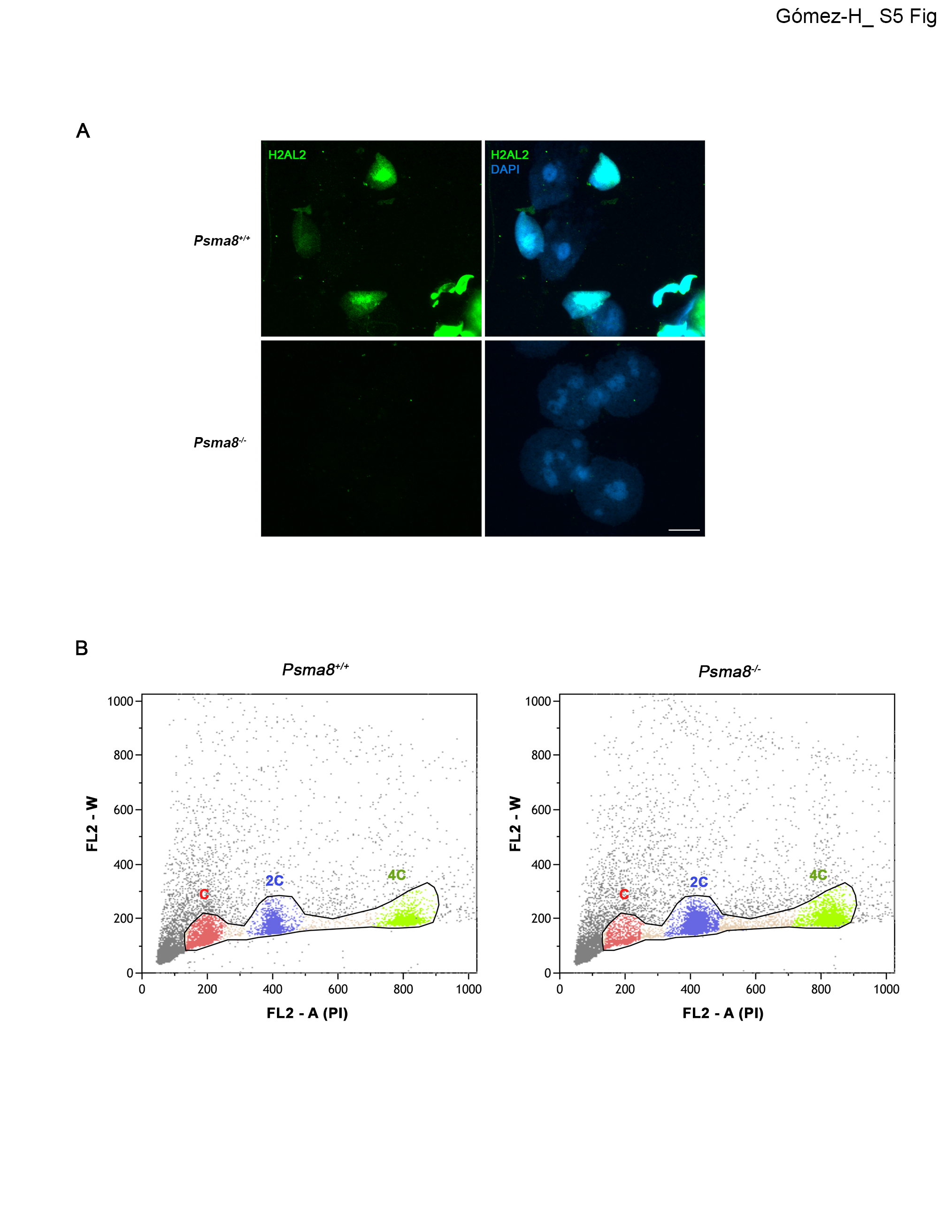

Supplement: S5 Fig — (A) Immunolabeling of H2AL2 (green) show positive staining in elongating spermatids from wild type mice but lack of staining in Psma8-/- mice. Chromatin was stained with DAPI. Bar represents 10 μm. (B) Gating strategy employed in the FACs analysis of Fig 3D. Grey dots represent cells that were excluded from the analysis whilst dots included in the polygon represent cells that were employed for the analysis. Red dots enclose 1C cells, blue dots represent 2C cells and green dots enclose 4C cells. (TIF) [file pgen.1008316.s005.tif]

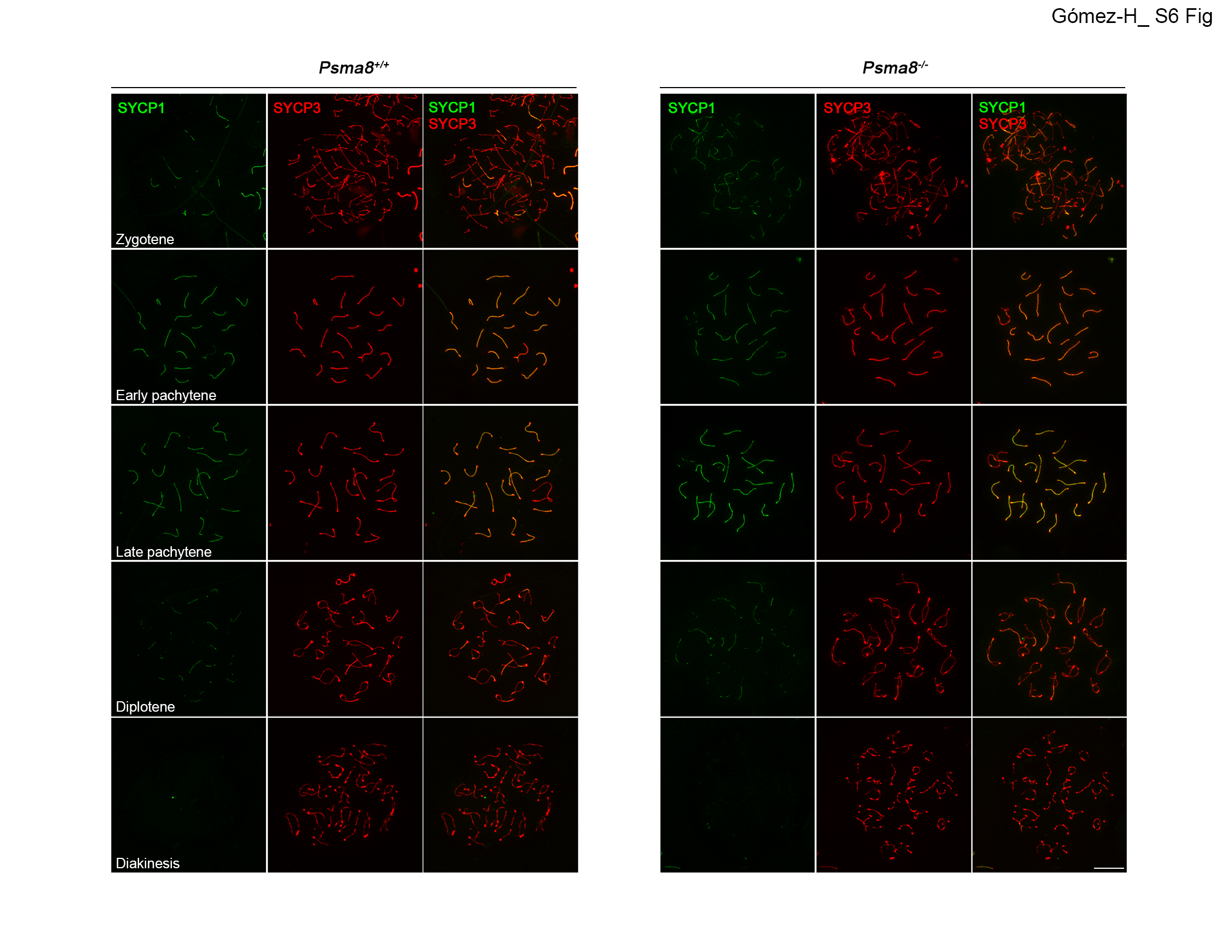

Supplement: S6 Fig — Double immunolabeling of SYCP3 (red) and SYCP1 (green) showing normal synapsis and desynapsis from early zygotene to diakinesis in Psma8-/- in comparison with Psma8+/+. Bar represents 10 μm. (TIF) [file pgen.1008316.s006.tif]

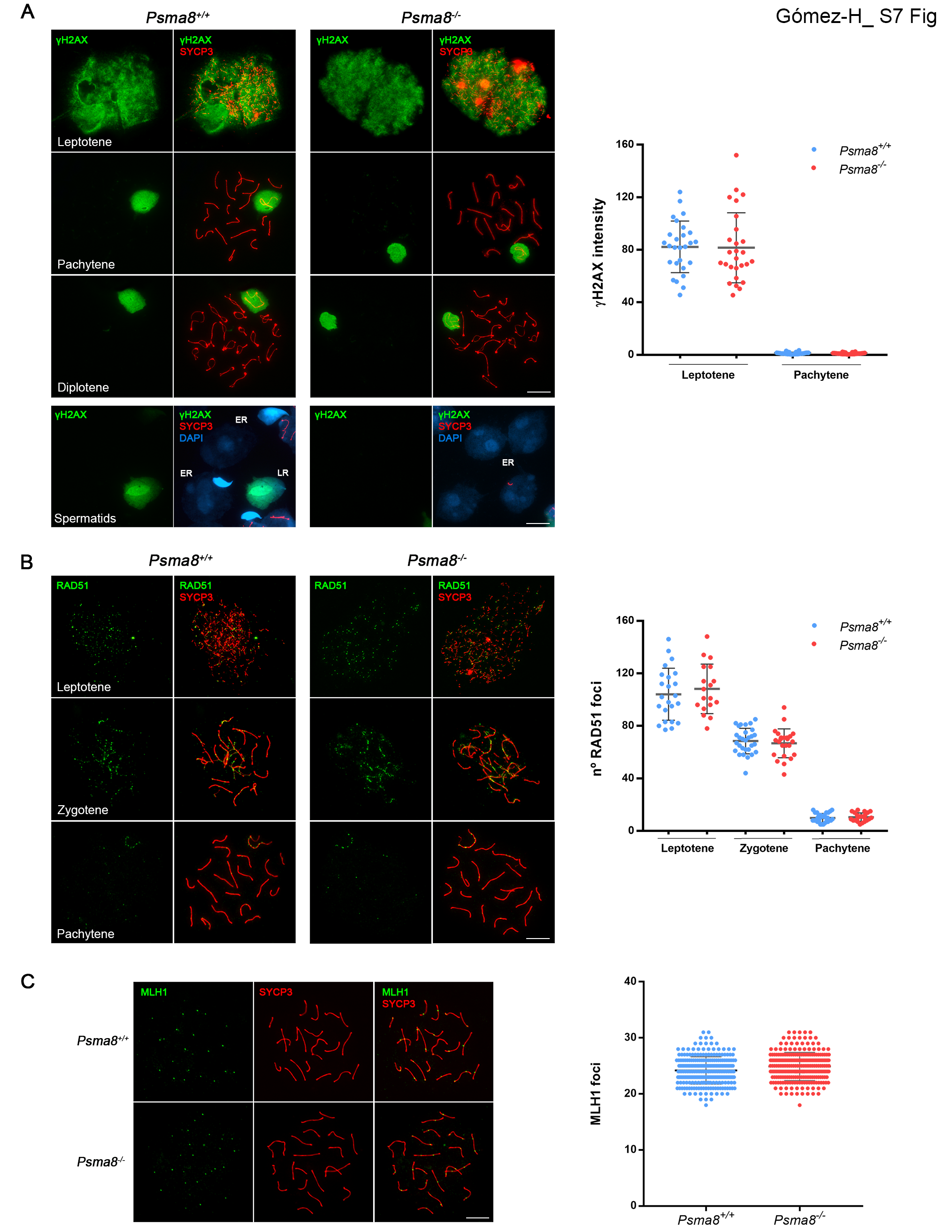

Supplement: S7 Fig — (A) Double immunolabeling of γ-H2AX (green) with SYCP3 (red) in wild-type and Psma8-/- spermatocytes from leptotene to diplotene (upper panel). In WT and KO leptonemas, γ-H2AX labels intensely the chromatin. After repair, γ-H2AX labeling remains only in the chromatin of the sex body of the pachynemas. Plot right to the panel represent the quantification of the fluorescence intensity from Psma8+/+ and Psma8-/- spermatocytes at leptotene and pachytene. Late round spermatids (LR) but not early round spermatids (ER) from wild type mice show positive staining for γ-H2AX but these highly differentiated cells are lacking in the Psma8-/- tubules which are arrested at early round spermatids without γ-H2AX staining (bottom panel). (B) Double immunolabeling of SYCP3 (red) and RAD51 (green). RAD51 foci associates to the AEs in leptonema spermatocytes of both genotypes (similar number of foci) and dissociate towards pachytene with a similar kinetics. Plot right to the image panel represents the quantification of the number of foci from Psma8+/+ and Psma8-/- spermatocytes. (C) Double immunolabeling of SYCP3 (red) with MLH1 (green). MLH1 foci are present along each autosomal SC in wild-type and Psma8-/- pachynema meiocytes in a similar way. Plot right to the panel represents the quantification of the values of the MLH1 foci from Psma8+/+ and Psma8-/- spermatocytes. Bars represent 10 μm. Welch´s t-test analysis: * p<0.01; ** p<0.001; *** p<0.0001. Quantification data is indicated in S3 Table. (TIF) [file pgen.1008316.s007.tif]

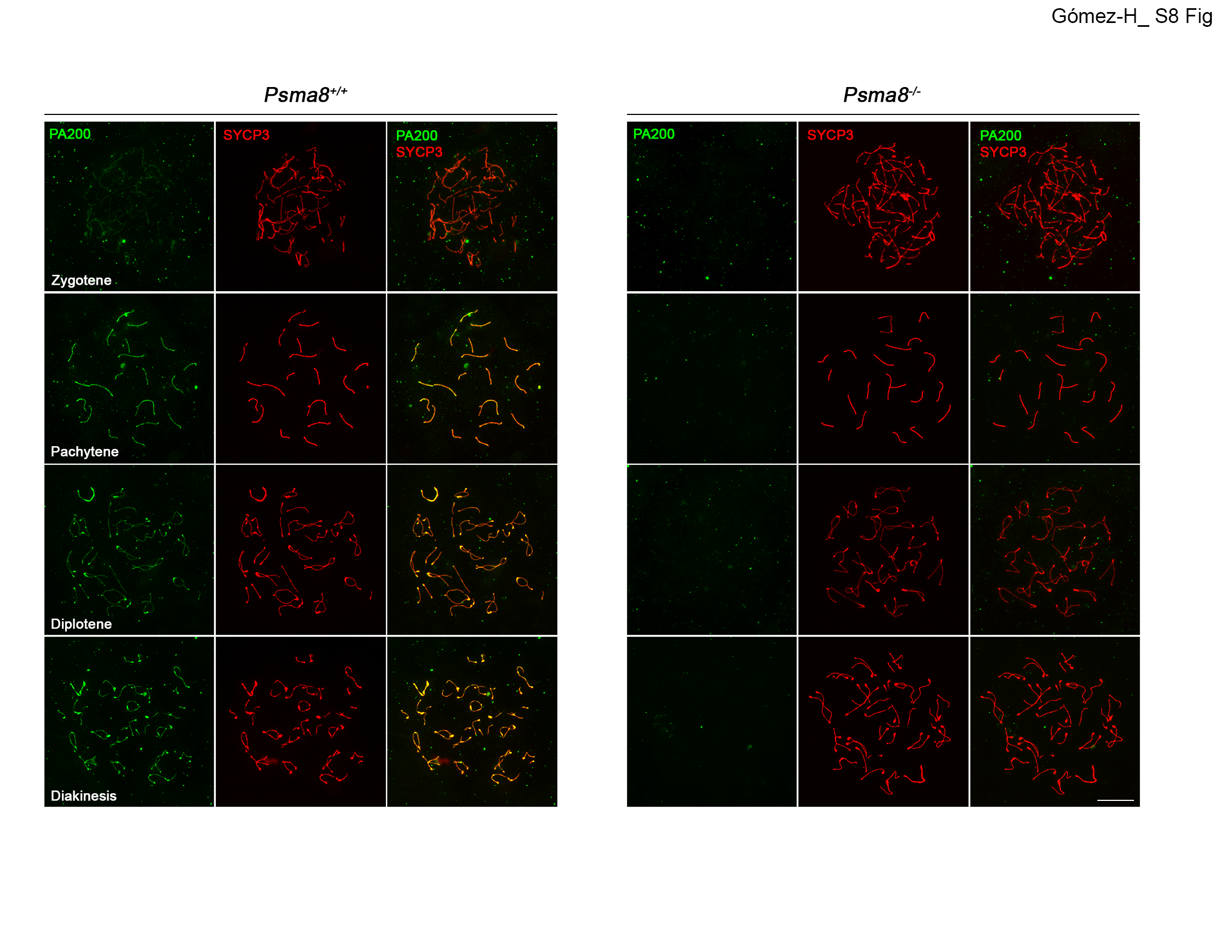

Supplement: S8 Fig — Double immunolabeling of PA200 (green) and SYCP3 (red) in chromosome spreads from zygotene to diakinesis. PA200 is detected at the chromosome axes in wild type spermatocytes in contrast to the absence of labeling in Psma8-/- spermatocytes. Bar in panels, 10 μm. (TIF) [file pgen.1008316.s008.tif]

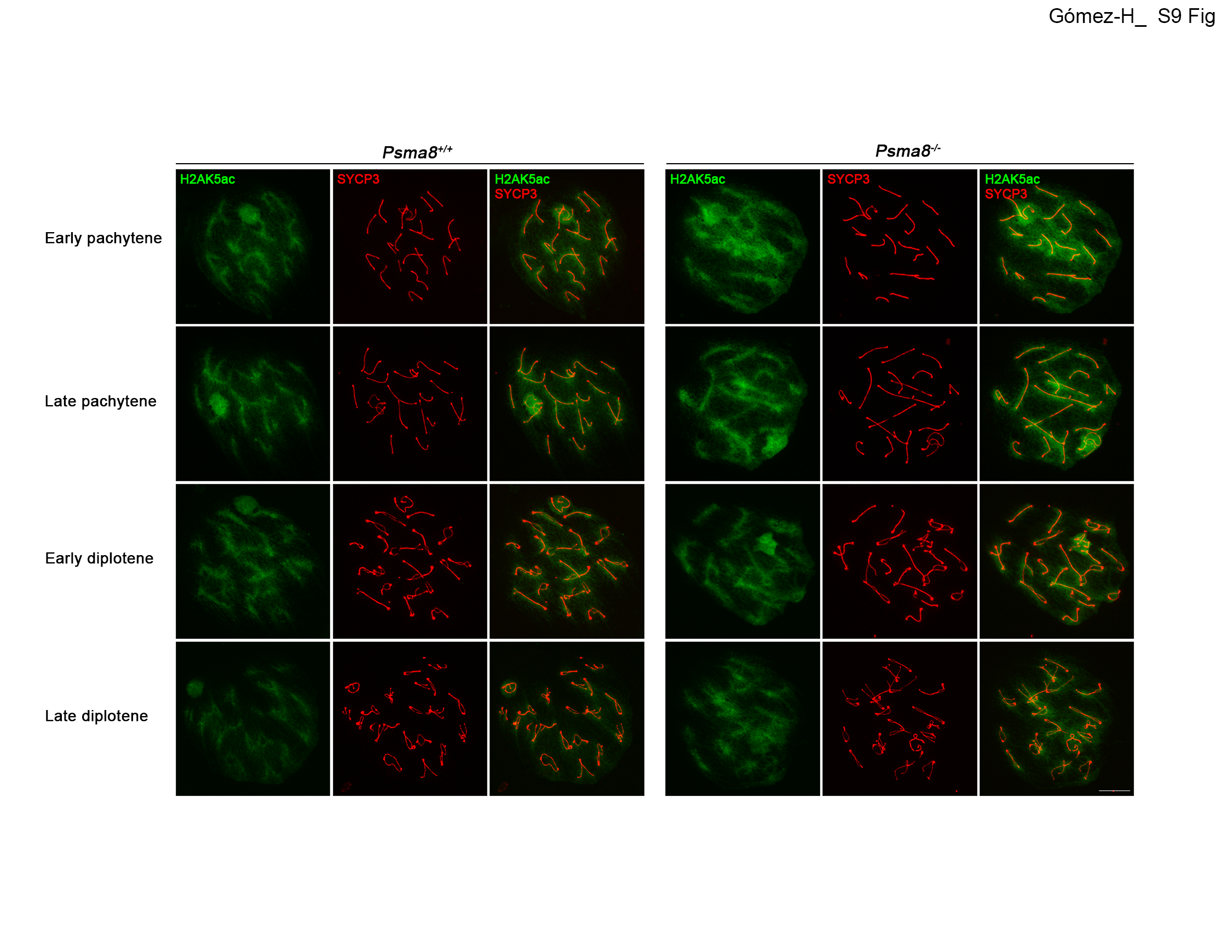

Supplement: S9 Fig — Double immunolabeling of H2AK5ac (green) with SYCP3 (red) in wild-type (left panel) and Psma8-/- spermatocytes (right panel). In WT and KO spermatocytes chromatin start to be labelled at early pachytene around chromosomes axes. Plots from each panel representing the quantification of fluorescence intensity from Psma8+/+ and Psma8-/- spermatocytes are depicted in Fig 4A. Bar represents 10 μm. (TIF) [file pgen.1008316.s009.tif]

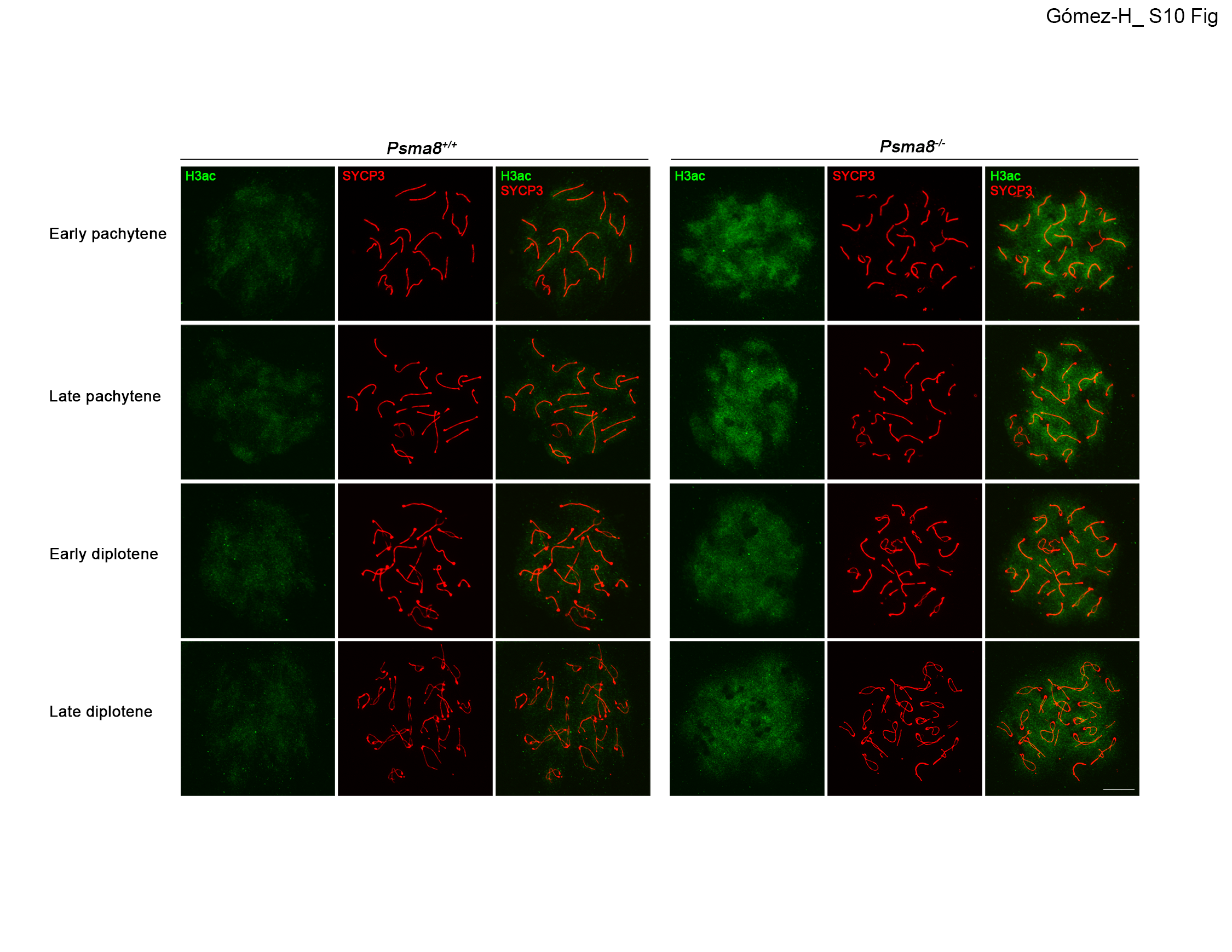

Supplement: S10 Fig — Double immunolabeling of H3ac (green) with SYCP3 (red) in wild-type (left panel) and Psma8-/- spermatocytes (right panel). Spermatocytes from Psma8+/+ and Psma8-/- show labeling for H3ac at early pachytene in a very diffuse manner surrounding chromosomes axes. Plots from each panel representing the quantification of fluorescence intensity from Psma8+/+ and Psma8-/- spermatocytes are in Fig 4B. Bar represents 10 μm. (TIF) [file pgen.1008316.s010.tif]

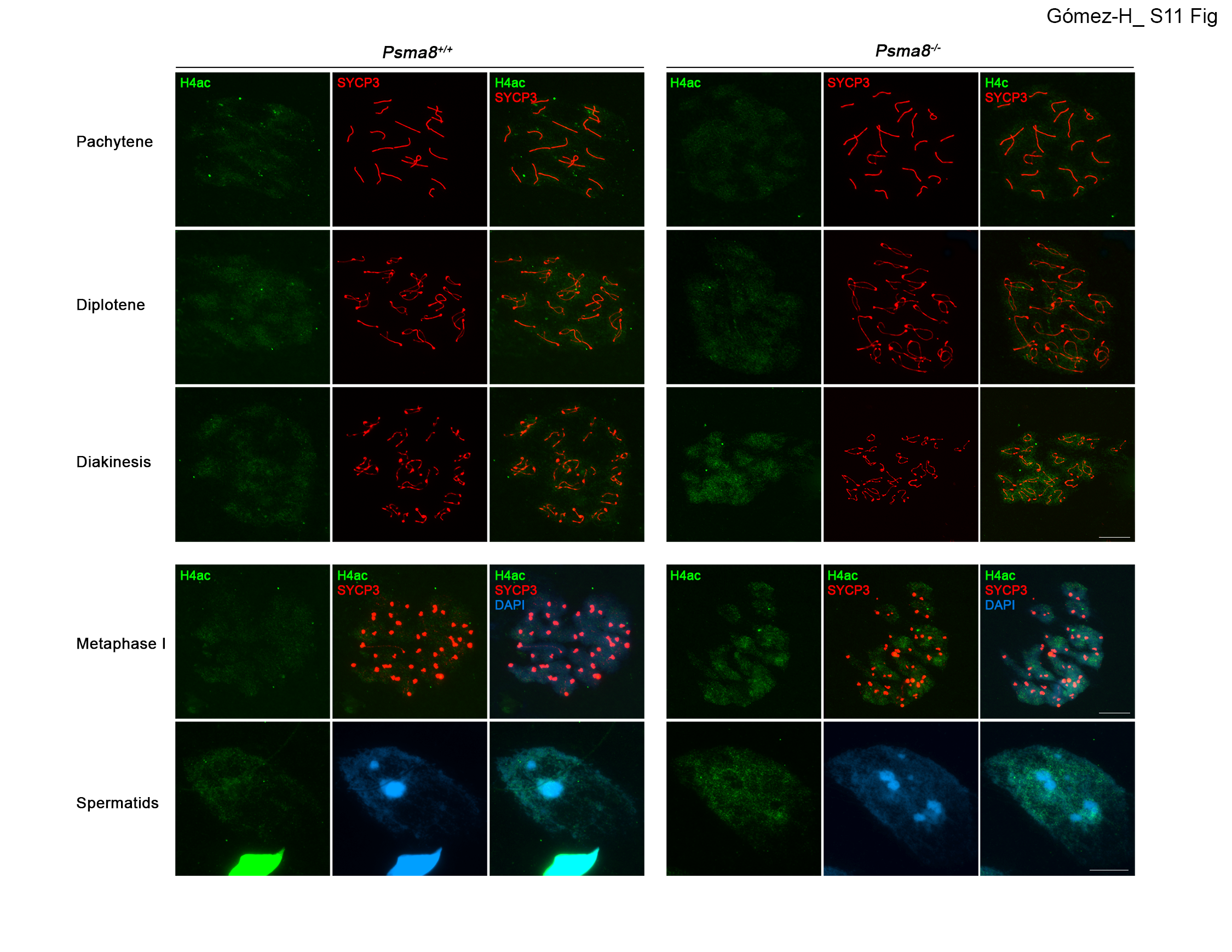

Supplement: S11 Fig — Double immunolabeling of H4ac (green) with SYCP3 (red) in wild-type and Psma8-/- spermatocytes. Spermatocytes from Psma8+/+ and Psma8-/- show labeling for H4ac in a very diffuse manner surrounding chromosomes from pachytene to metaphase I (right panel). In wild type metaphase I, H4ac labeling appears weakly painting the chromosomes and on some of the centromeres. However, Psma8-deficient cells show a more intense labeling specially at the centromeres (lower panel). Round spermatid from Psma8-/- accumulates H4ac labeling at the chromatin in comparison with the WT. Plots from each panel representing the quantification of fluorescence intensity from Psma8+/+ and Psma8-/- spermatocytes are in Fig 4C. Bars represent 10 μm. (TIF) [file pgen.1008316.s011.tif]

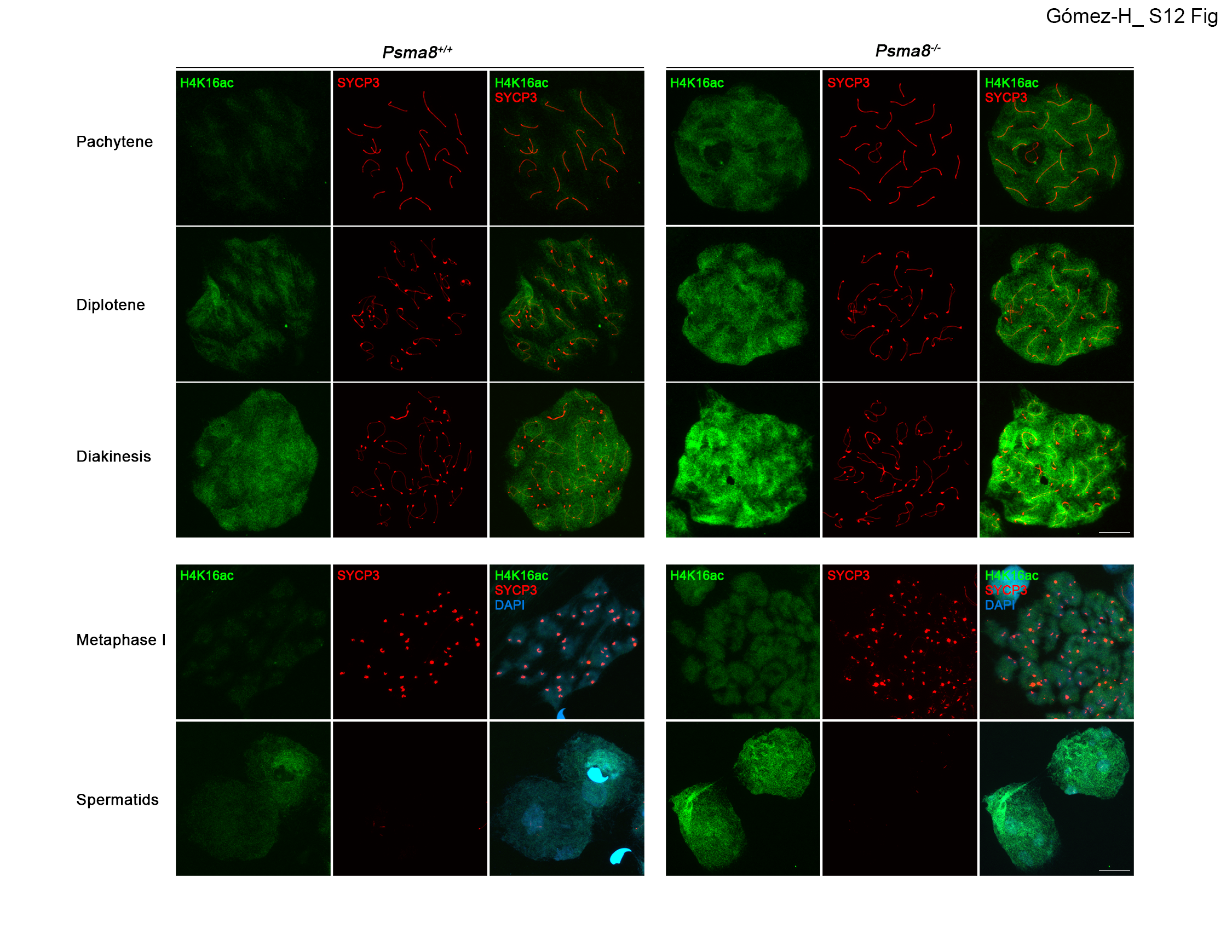

Supplement: S12 Fig — Double immunolabeling of H4K16ac (green) with SYCP3 (red) in wild-type and Psma8-/- spermatocytes. Spermatocytes from Psma8+/+ and Psma8-/- show labeling for H4K16ac in a very diffuse manner surrounding chromosomes from pachytene to metaphase I (right panel). In wild type metaphase I, H4K16ac labeling appears weakly painting the chromosomes. However, Psma8-deficient cells show enhance labeling in the chromosomes of metaphase I cells (lower panel). Round spermatid from Psma8-/- accumulates H4K16ac labeling at the chromatin in comparison with the WT. Plots from each panel representing the quantification of fluorescence intensity from Psma8+/+ and Psma8-/- spermatocytes are in Fig 4D. Bars represent 10 μm. (TIF) [file pgen.1008316.s012.tif]

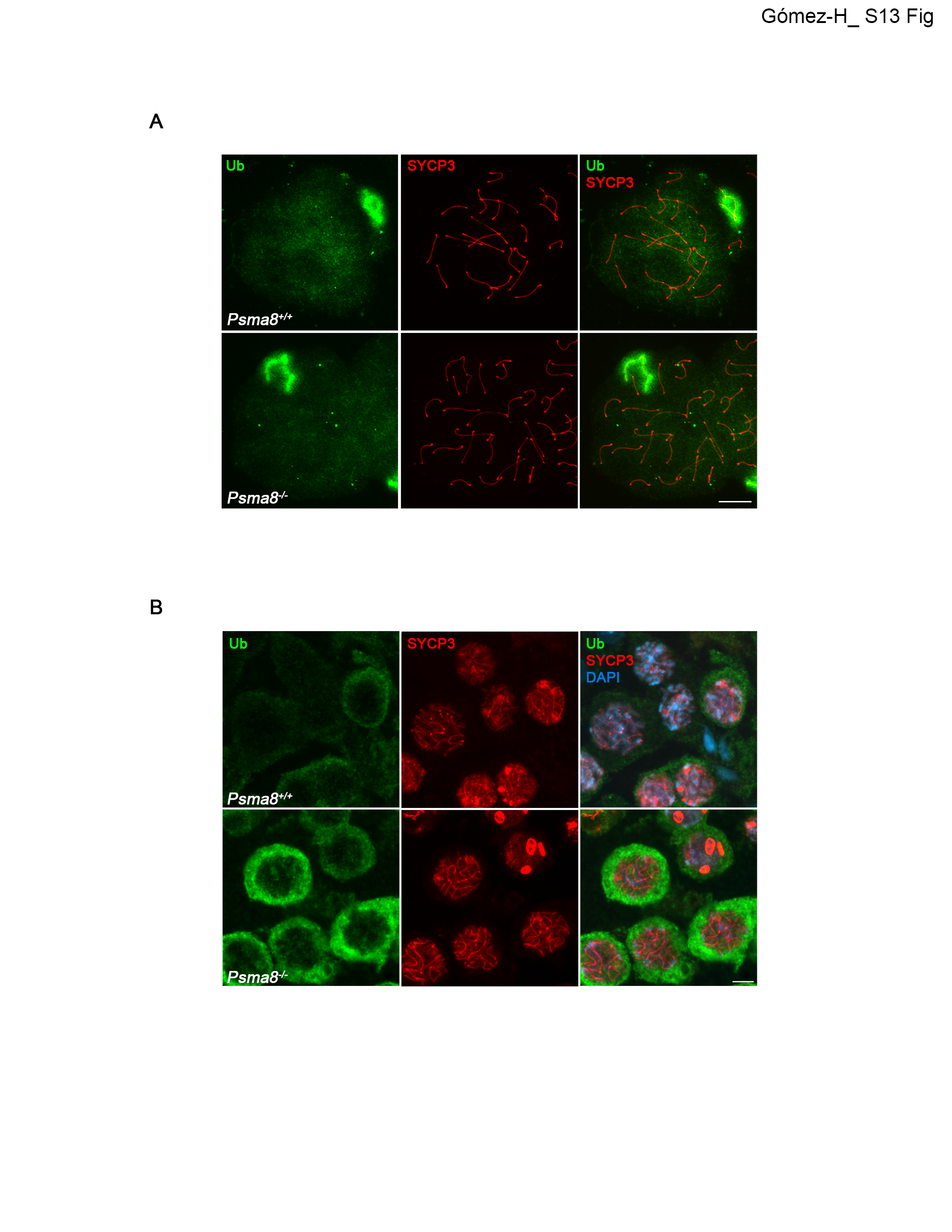

Supplement: S13 Fig — (A) Double immunolabeling of Ubiquitin (green) and SYCP3 (red) in mouse chromosome spreads at pachytene stage from Psma8+/+ and Psma8-/-mice. (B) Double immunolabeling of Ubiquitin (green) and SYCP3 (red) in mouse squashed tubules from Psma8+/+ and Psma8-/- mice. Chromatin was stained with DAPI. Bars represent 10 μm (A) and 5 μm (B). (TIF) [file pgen.1008316.s013.tif]

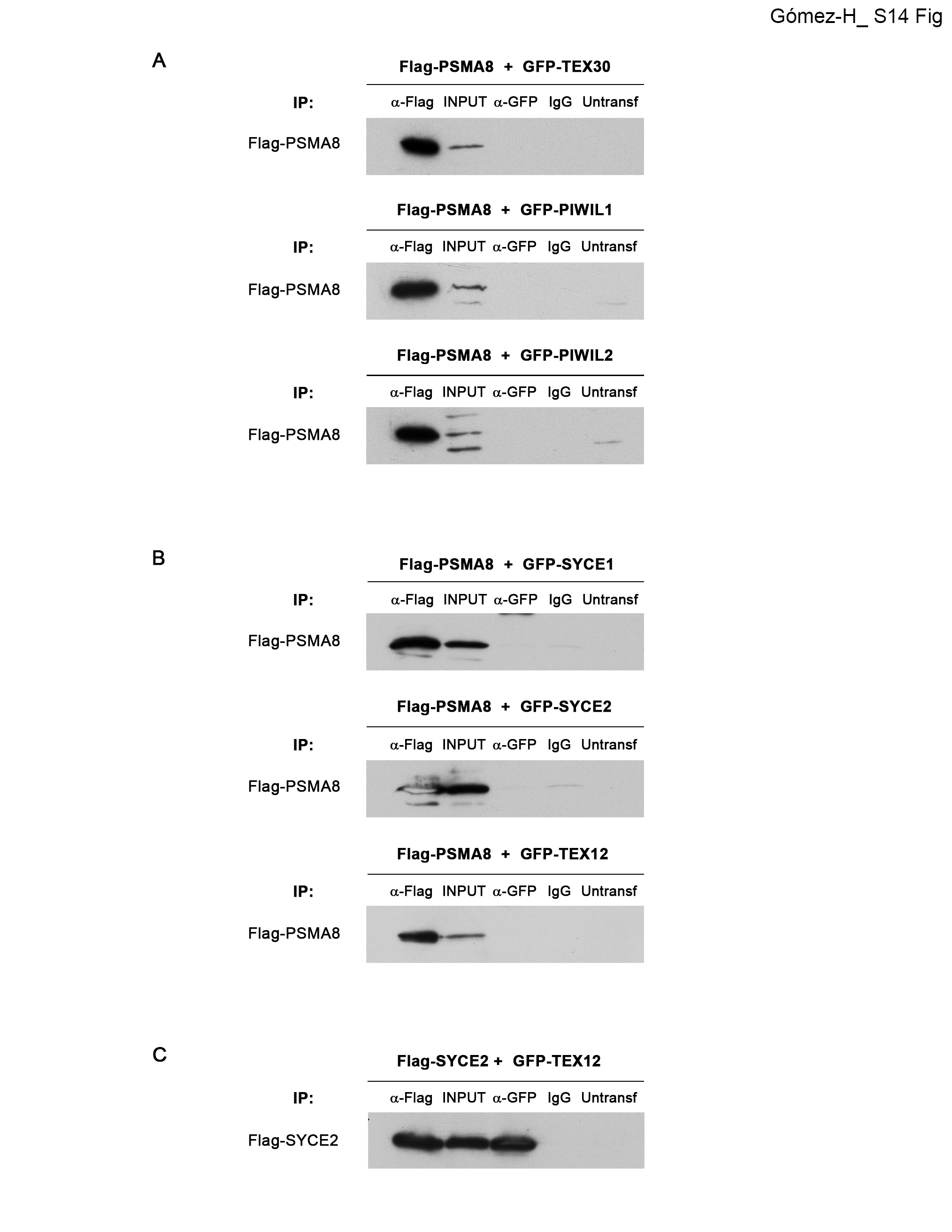

Supplement: S14 Fig — (A-B) HEK293T cells were co-transfected with GFP-TEX30, GFP-PIWIL1, GFP-PIWIL2, GFP-SYCE1, GFP-SYCE2, and GFP-TEX12, and with Flag-PSMA8. PSMA8 does not co-immunoprecipitates (co-IP) with any of them. (C) Positive control was generated by transfecting HEK293T cells with Flag-SYCE2 and GFP-TEX12. Protein complexes were immunoprecipitated overnight with either an anti-Flag or anti-EGFP or IgGs (negative control) and were analyzed by immunoblotting with the indicated antibody. (TIF) [file pgen.1008316.s014.tif]

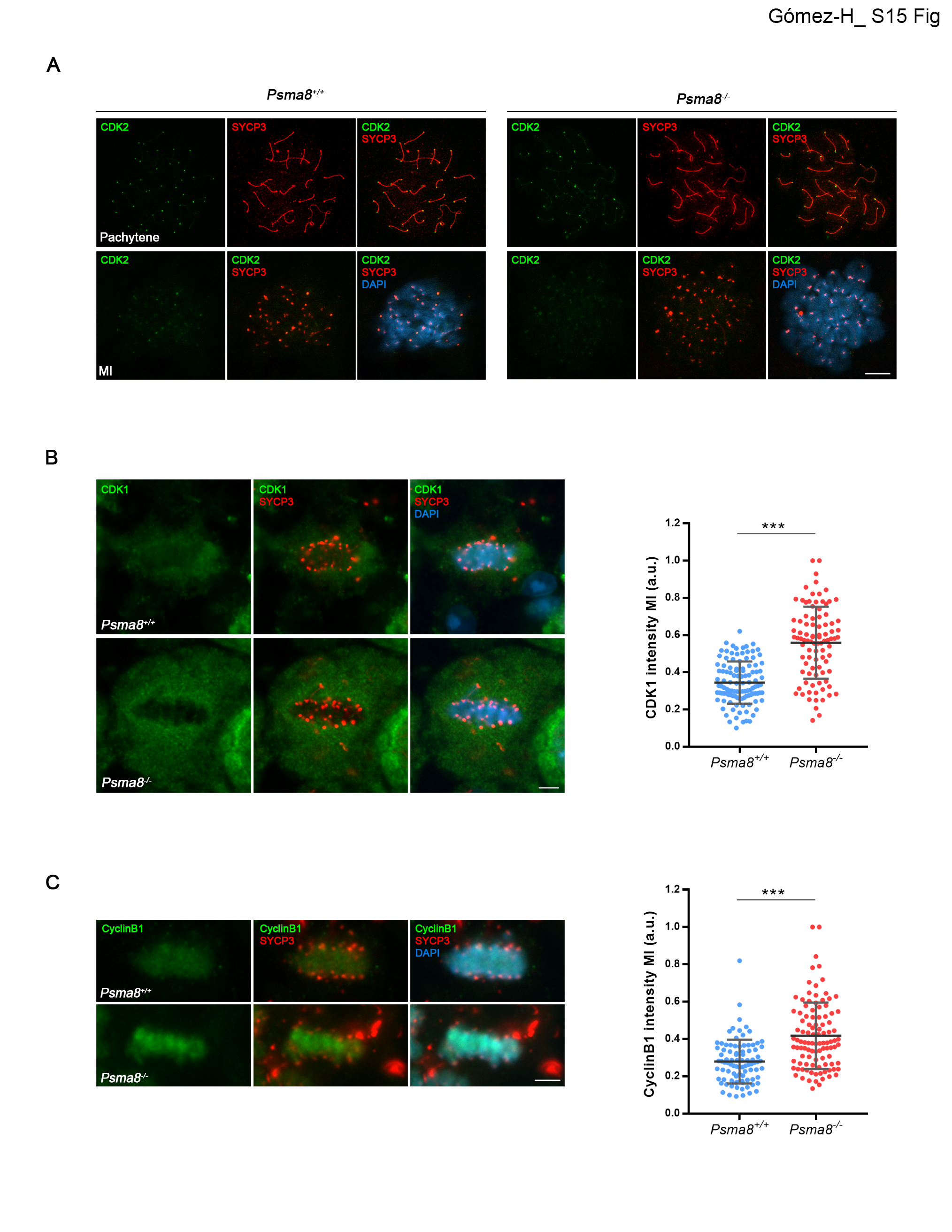

Supplement: S15 Fig — (A) Double immunolabeling of endogenous CDK2 (green) and SYCP3 (red) in WT and KO mouse chromosome spreads at pachytene and metaphase I showing similar labeling at the telomeres and centromeres, respectively. (B) Double immunolabeling of CDK1 (green) and SYCP3 (red) in mouse squashed metaphases I from Psma8+/+ and Psma8-/-mice showing CDK1 accumulation. Plot right to the panel represents the quantification of total CDK1 fluorescence intensity from Psma8+/+ and Psma8-/- metaphase I cells. (C) Double immunolabeling of Cyclin B1 (green) and SYCP3 (red) in mouse squashed tubules from Psma8+/+ and Psma8-/- mice showing CyclinB1 accumulation. Plot right to the panel represents the quantification of total CyclinB1 fluorescence intensity in metaphase I cells. Bars represent 10 μm (A), and 5 μm (B,C). Welch´s t-test analysis: * p<0.01; ** p<0.001; *** p<0.0001. (TIF) [file pgen.1008316.s015.tif]

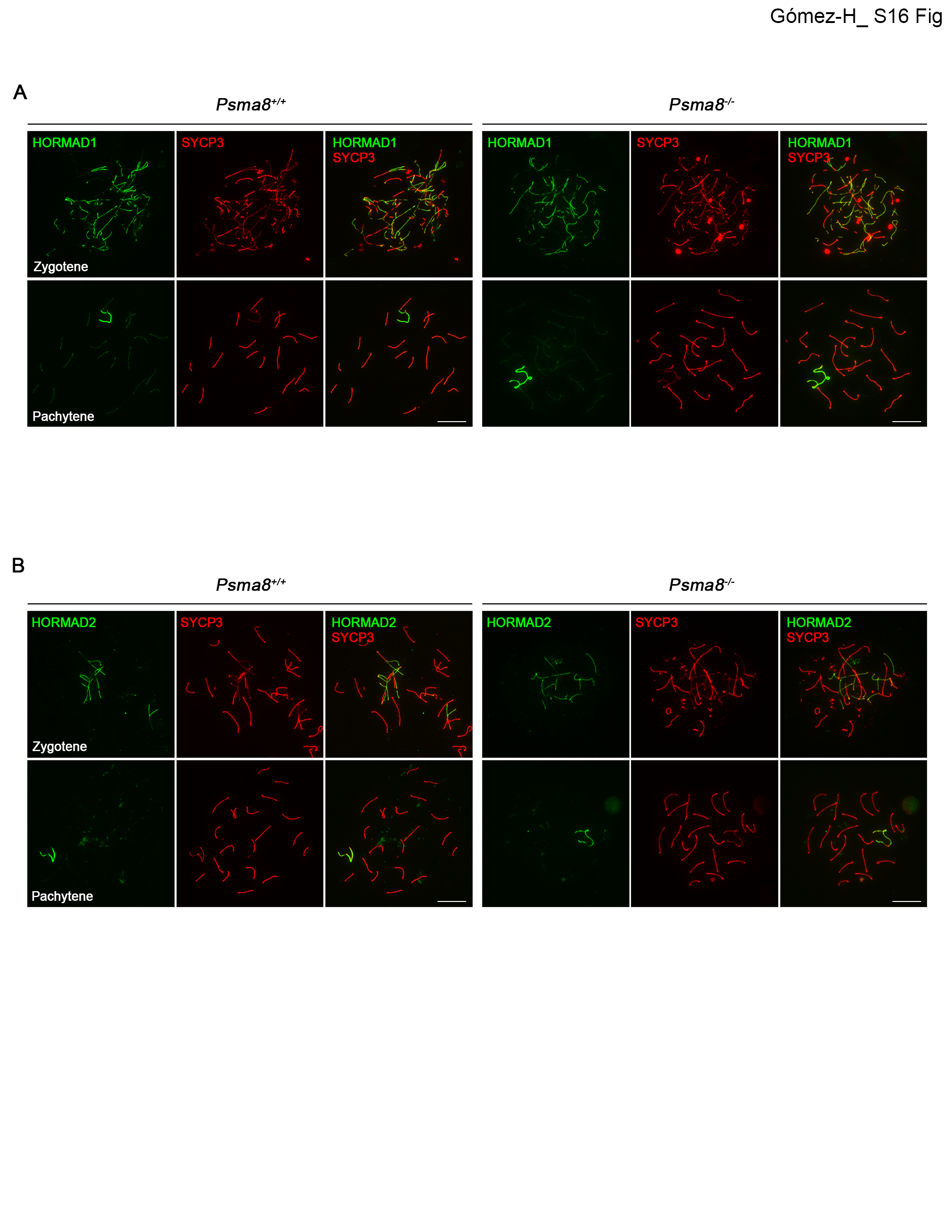

Supplement: S16 Fig — (A-B) Double immunolabeling of HORMAD1 (A) and HORMAD2 (B) (green) with SYCP3 (red) in Psma8+/+ and Psma8-/- spermatocytes at zygotene and pachytene stages. As synapsis progresses HORMAD1 and HORMAD2 are released from the AEs and maintained at the AE of the sex body similarly in the wild type and in the mutant spermatocytes. Bars represent 10 μm. (TIF) [file pgen.1008316.s016.tif]

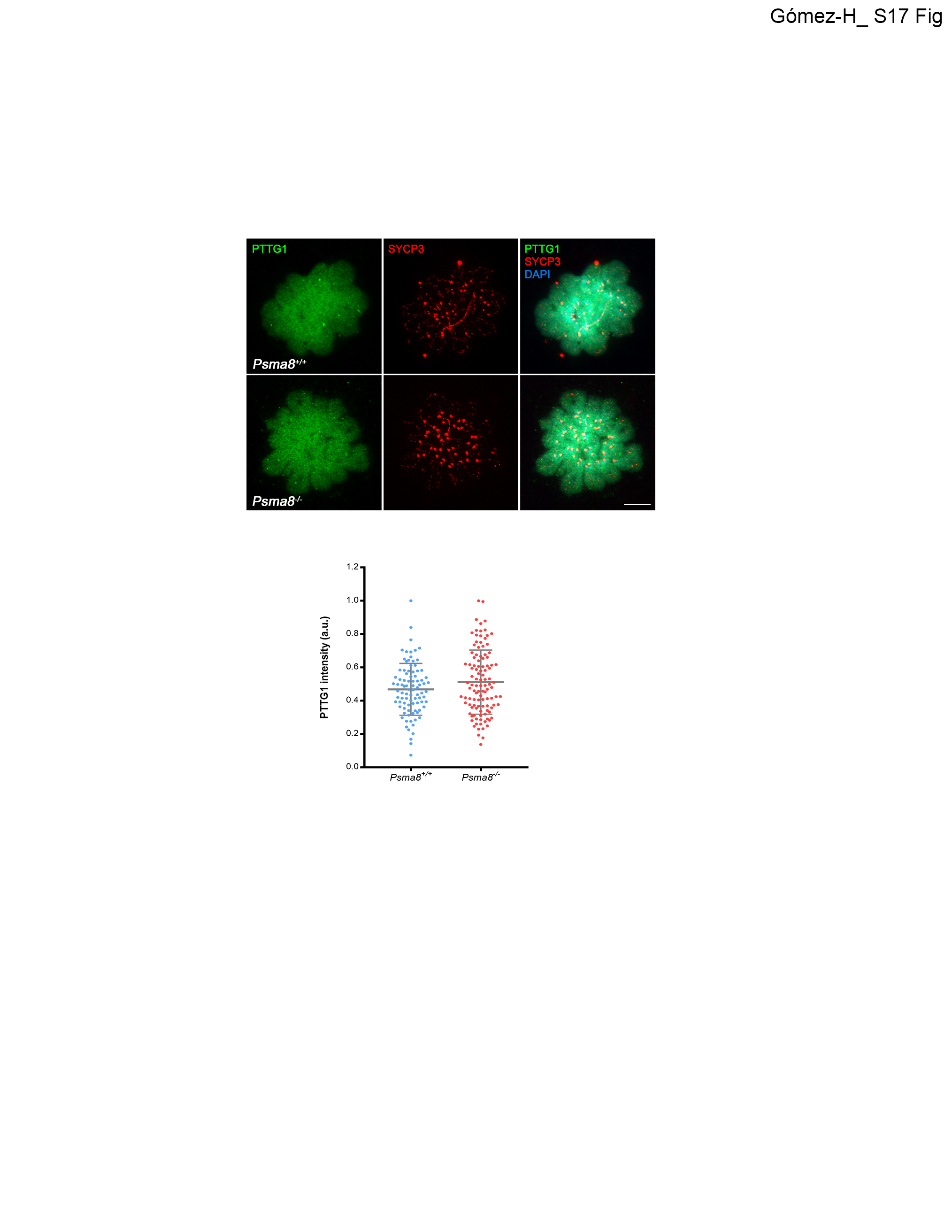

Supplement: S17 Fig — Double immunofluorescence of PTTG1 (green) and SYCP3 (red) in metaphase I cells showing similar expression levels of PTTG1. Plot under the panel represents the quantification of the fluorescence intensity from Psma8+/+ and Psma8-/- metaphase I cells. Bar in panels, 10 μm. Welch´s t-test analysis: * p<0.01; ** p<0.001; *** p<0.0001. (TIF) [file pgen.1008316.s017.tif]

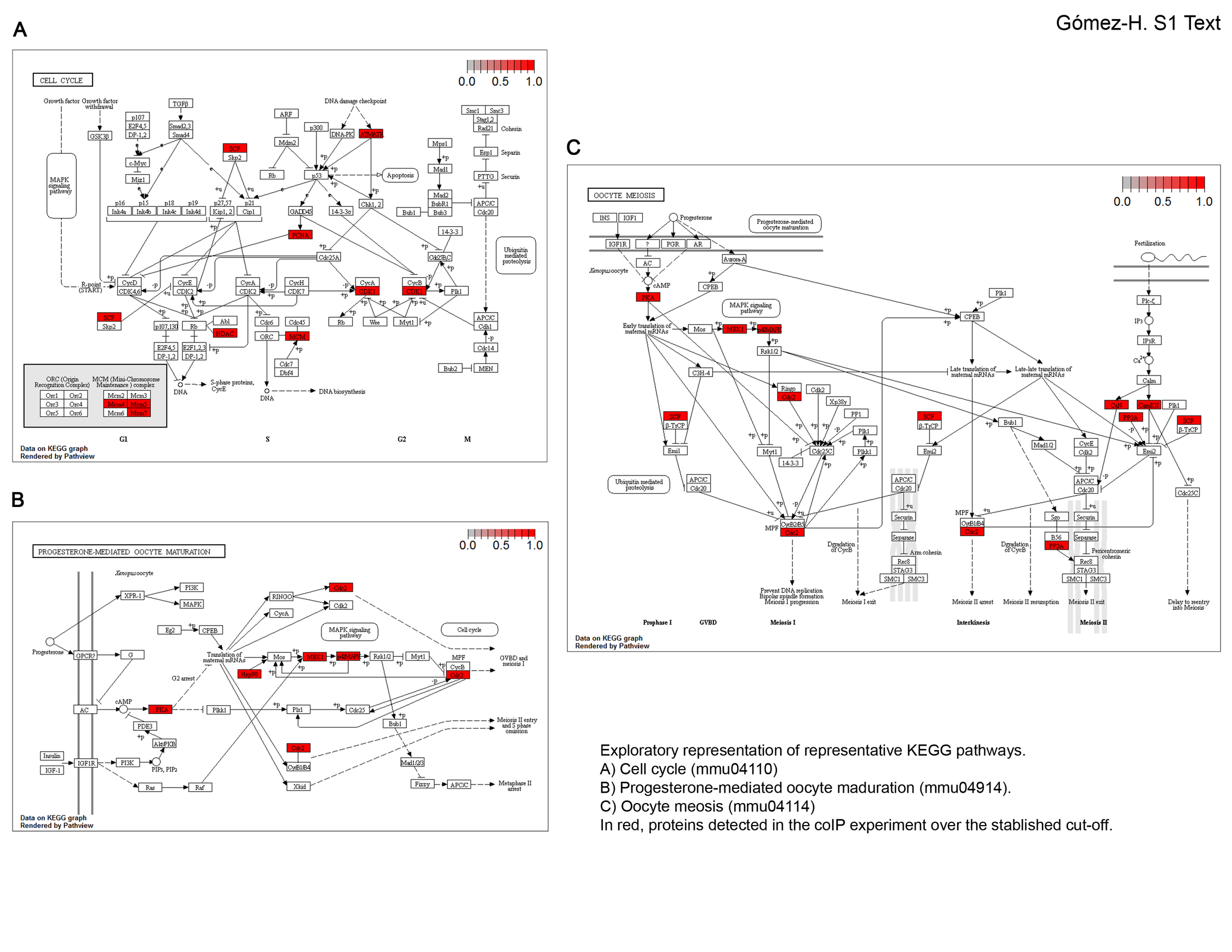

Supplement: S1 Text — (A) Cell cycle (mmu04110). (B) Progesterone-mediated oocyte maduration (mmu04914). (C) Oocyte meiosis (mmu04114). In red, proteins detected in the co-IP experiment over the established cut-off. (HTM) [file pgen.1008316.s025.htm]
