# Supplementary material for: The PSMA8 subunit of the spermatoproteasome is essential for proper meiotic exit and mouse fertility
Source: PLoS Genet. 2019 Aug 22;15(8):e1008316. doi: 10.1371/journal.pgen.1008316 (PMC6726247; doi:10.1371/journal.pgen.1008316)
Supplement: S1 Table — (PDF) [file pgen.1008316.s018.pdf]

S1 Table. Fertility assessment of *Psma8*$^{+/+}$, *Psma8*$^{+/-}$ and *Psma8*$^{-/-}$ mice.

Male	Female	nº litters	nº pups
Psma8$^{+/-}$	***Psma8***$^{+/-}$	40	6.45 ± 2.24
Psma8$^{-/-}$	***Psma8***$^{+/+}$	0	0 ± 0
Psma8$^{+/+}$	***Psma8***$^{-/-}$	2	6.50 ± 0.71
Psma8$^{+/-}$	***Psma8***$^{-/-}$	25	8.28 ± 2.17
