# Supplementary material for: The PSMA8 subunit of the spermatoproteasome is essential for proper meiotic exit and mouse fertility
Source: PLoS Genet. 2019 Aug 22;15(8):e1008316. doi: 10.1371/journal.pgen.1008316 (PMC6726247; doi:10.1371/journal.pgen.1008316)
Supplement: S2 Table — (A) Quantification of the proportion of tubules with metaphase I/II in PAS stained tubule sections from the histology example shown in Fig 2B. (B) Quantification of the number of metaphase I and II cells present in p-Ser10-H3 stained tubules that show meiotic divisions (Fig 2C). (C) Quantification of the percentage of metaphases-anaphases I and metaphases-anaphases II in squash preparations (double immunolabeled with ACA and SYCP3) measured as the N° of Metaphase-Anaphase I/II divided by the N° of cells (prophase I + Metaphase-Anaphase I + Interkinesis +Metaphase-Anaphase II) (Fig 2D). Apoptotic Metaphase-Anaphase I and Metaphase-Anaphase II within each genotype are indicated. (PDF) [file pgen.1008316.s019.pdf]

**S2 Table. Quantification of metaphases I/II in *Psma8$^{-/-}$* testis.**

(A) Quantification of the the proportion of tubules with metaphase I/II in PAS stained tubule sections from the histology example shown in Fig 2B. (B) Quantification of the number of metaphase I and II cells present in p-Ser10-H3 stained tubules that show meiotic divisions (Fig 2C). (C) Quantification of the percentage of metaphases-anaphases I and metaphases-anaphases II in squash preparations (double immunolabeled with ACA and SYCP3) measured as the Nº of Metaphase-Anaphase I/II divided by the Nº of cells (prophase I + Metaphase-Anaphase I + Interkinesis +Metaphase-Anaphase II) (Fig 2D). Apoptotic Metaphase-Anaphase I and Metaphase-Anaphase II within each genotype are indicated.

## A

| | n (mice) | Tubules with meiotic divisions | Other stages | Total tubules | % meiotic divisions | Mean (%) |
|---|---|---|---|---|---|---|
| WT | 1 | 17 | 362 | 379 | 4.5 | 5.37 ± 1.50 |
| | 2 | 32 | 419 | 451 | 7.1 | |
| | 3 | 27 | 569 | 596 | 4.5 | |
| KO | 1 | 51 | 377 | 428 | 12.6 | 12.50 ± 0.10 |
| | 2 | 50 | 353 | 403 | 12.4 | |
| | 3 | 46 | 323 | 369 | 12.5 | |

## B

| | % tubules | |
|---|---|---|
| Nº cells pH3 positive | WT | KO |
| 0 | 77.22 | 54.36 |
| 1- 4 cells | 16.11 | 23.49 |
| 5-9 cells | 2.78 | 10.07 |
| ≥ 10 cells | 3.89 | 12.08 |
| n (tubules) | 180 | 149 |

## C

| | MI - AI | | MII - AII | |
|---|---|---|---|---|
| | WT | KO | WT | KO |
| Total | 1.66 ± 0.50 | 6.75 ± 0.52 | 0.92 ± 1.03 | 8.32 ± 3.35 |
| Apoptotic | 0.09 ± 0.13 | 3.90 ± 0.53 | 0.00 ± 0.00 | 6.17 ± 4.05 |
