# Supplementary material for: The PSMA8 subunit of the spermatoproteasome is essential for proper meiotic exit and mouse fertility
Source: PLoS Genet. 2019 Aug 22;15(8):e1008316. doi: 10.1371/journal.pgen.1008316 (PMC6726247; doi:10.1371/journal.pgen.1008316)
Supplement: S3 Table — (PDF) [file pgen.1008316.s020.pdf]

S3 Table. Quantification of γH2AX levels, RAD51 foci, and MLH1 foci (S7 Fig).

γH2AX		Mean (intensity)	SD	n
Leptotene	WT	82.23	19.64	27
	KO	81.58	26.69	27
Pachytene	WT	1.33	0.79	28
	KO	1.17	0.59	28

RAD51		Nº foci	SD	n
Leptotene	WT	104.13	19.80	23
	KO	111.53	18.79	18
Zygotene	WT	65.52	9.69	29
	KO	66.79	10.98	24
Pachytene	WT	9.90	3.39	29
	KO	10.55	3.23	22

MLH1		Nº foci	SD	n	Mean
WT	1	23.78	2.64	51	24.35 ± 1.12
	2	23.07	2.12	94	
	3	25.07	2.03	41	
	4	25.48	1.91	67	
KO	1	26.03	2.80	62	24.90 ± 0.94
	2	24.11	2.00	85	
	3	24.13	1.94	39	
	4	25.32	2.66	60	
