# Supplementary material for: The PSMA8 subunit of the spermatoproteasome is essential for proper meiotic exit and mouse fertility
Source: PLoS Genet. 2019 Aug 22;15(8):e1008316. doi: 10.1371/journal.pgen.1008316 (PMC6726247; doi:10.1371/journal.pgen.1008316)
Supplement: S4 Table — (PDF) [file pgen.1008316.s021.pdf]

S4 Table: Proteasome subunits and proteasome regulators co-immunoprecipitated with PSMA8 from *Psma8*$^{+/+}$ and *Psma8*$^{-/-}$ testis protein extracts using anti-PSMA8 R2 antibody.

Name	Uniprot Accession ID	Nº of unique peptides			iBAQ Intensity			
		WT	KO	IgG	WT	KO	IgG	Ratio WT/KO
**20 S Proteasome subunits**								
PSMA1 (α1)	Q9R1P4	4	1	0	1213200	6249.7	0	194.1
PSMA2 (α2)	P49722	1	0	0	163770	0	0	∞
PSMA3 (α3)	O70435	4	2	2	1215300	52164	216190	23.3
PSMA4 (α4)	Q9R1P0	2	0	3	452740	0	55451	∞
PSMA5 (α5)	Q9Z2U1	5	1	1	1107800	76592	111540	14.5
PSMA6 (α6)	Q9QUM9	2	3	2	354100	126040	154580	2.8
PSMA7 (α7)	Q9Z2U0	6	2	1	11121000	509900	32808	21.8
PSMA8 (α4s)	Q9CWH6	6	2	2	120340000	892150	359850	134.9
PSMB1 (β1)	O09061	4	2	3	1344300	58017	153670	23.2
PSMB2 (β2)	Q9R1P3	2	1	0	113520	20656	0	5.5
PSMB3 (β3)	Q9R1P1	4	0	2	853790	0	56617	∞
PSMB4 (β4)	P99026	2	0	0	263710	0	0	∞
PSMB5 (β5)	O55234	6	2	2	1387200	66683	115990	20.8
PSMB6 (β6)	Q60692	1	0	0	44701	0	0	∞
PSMB7 (β7)	P70195	1	0	0	218870	0	0	∞
**Proteasome activators**								
PSME3 (PA28γ)	A2A4J1	0	1	0	0	33185	0	0.0
PSME4 (PA200)	Q5SSW2	1	0	0	3386.8	0	0	∞
