# Supplementary material for: The PSMA8 subunit of the spermatoproteasome is essential for proper meiotic exit and mouse fertility
Source: PLoS Genet. 2019 Aug 22;15(8):e1008316. doi: 10.1371/journal.pgen.1008316 (PMC6726247; doi:10.1371/journal.pgen.1008316)
Supplement: S5 Table — (PDF) [file pgen.1008316.s022.pdf]

S5 Table: Proteasome subunits and proteasome regulators co-immunoprecipitated with PSMA8 selected after analysis and filtering of the data.

Name	Uniprot Accession ID	No of unique peptides			Sequence coverage			iBAQ Intensity			Ref.
		Ab1	Ab2	Control	Ab1	Ab2	Control	Ab1	Ab2	Control	
**20 S Proteasome subunits**											
PSMA1 (α1)	Q9R1P4	8	4	0	35.7	15.6	0	21249000	1213200	0	A
PSMA2 (α2)	P49722	7	1	0	41.5	6	0	12548000	163770	0	A
PSMA3 (α3)	O70435	7	4	1	33.3	19.6	4.7	20726000	1215300	79317	A
PSMA4 (α4)	Q9R1P0	7	2	1	38.7	10	3.8	29307000	452740	54041	A
PSMA5 (α5)	Q9Z2U1	9	5	0	49.8	24.1	0	21477000	1107800	0	A
PSMA6 (α6)	Q9QUM9	7	2	1	35.4	10.2	4.1	9870500	354100	28488	A
PSMA7 (α7)	Q9Z2U0	4	6	0	46.8	52.4	5.6	11999000	11121000	0	A
PSMA8 (α4s)	Q9CWH6	9	6	1	62	43.2	8.8	259210000	120340000	176410	A
PSMB1 (β1)	O09061	13	4	1	61.2	21.7	5.8	35406000	1344300	69458	A
PSMB2 (β2)	Q9R1P3	5	2	0	35.8	12.9	0	6124800	113520	0	A
PSMB3 (β3)	Q9R1P1	7	4	1	41.5	26.3	7.8	26797000	853790	66486	A
PSMB4 (β4)	P99026	5	2	0	35.6	13.3	0	8425400	263710	0	A
PSMB5 (β5)	O55234	13	6	2	57.2	23.1	8.7	26458000	1387200	91924	A
PSMB6 (β6)	Q60692	3	1	0	12.6	3.8	0	6322100	44701	0	A
PSMB7 (β7)	P70195	2	1	0	11.6	3.6	0	1435700	218870	0	A
**Proteasome regulators**											
19 S subunits											
PSMC1 (S4)	P62192	9	5	1	28.4	16.4	2.7	1670700	342000	62230	A
PSMC2 (S7)	P46471	8	4	4	23.3	10.6	11.3	1372900	289950	147590	A
PSMC3 (S6a)	A2AGN7	7	4	4	25.2	12.2	17.8	1011500	126150	172160	A
PSMC4 (S6b)	A0A140LIZ5	7	2	2	22.5	4.9	4.9	710210	136800	55476	A
PSMC5 (S8)	P62196	5	8	1	16.5	24.6	2.7	674310	255690	33610	A
PSMC6 (S10b)	P62334	9	5	5	29.6	15.2	16.2	1830300	176350	145970	A
PSMD1 (Rpn2/S1)	Q3TXS7	9	2	2	14.5	2.3	3.3	288850	15023	26996	A
PSMD2* (RPN1/S2)	Q8VDM4	7	7	11	10.7	8.8	13.9	439090	144410	263690	A
PSMD3 (Rpn3/S3)	P14685	8	5	5	17.9	11.1	11.7	1333400	156560	129110	A
PSMD4* (Rpn10/S5A)	O35226	1	1	0	4.8	3.2	0	246070	35322	0	A
PSMD5 (S5B)	Q8BJY1	2	2	1	5.6	3.6	1.8	106410	66715	18125	A
PSMD6* (Rpn7/S10)	Q99JI4	1	1	0	3.1	2.6	0	71743	15485	0	A
PSMD11 (Rpn6/S9)	Q8BG32	4	2	2	10.9	4	5.5	220960	42386	31410	A
PSMD13 (Rpn9/S11)	E9Q5I9	0	2	0	0	5.2	0	0	42839	0	A
Psmd14* (Rpn11)	O35593	1	1	1	4.2	4.2	4.2	175390	96458	47666	A
Other activators											
PSME3 (PA28γ)	A2A4J1	2	0	1	14.8	0	7.4	236880	0	33185	A
PSME4 (PA200)	Q5SSW2	3	1	0	1.7	0.7	0	25422	3386.8	0	A
Substoichiometric proteasome protein											
TXNL1	Q8CDN6	2	3	1	10	10.7	2.4	359900	110780	17998	B

*Due to their relevance, these proteins were also included in the present table as an “ad-hoc” selection to show their behavior in spite of them not passing our cut-off.

A Wang, X., Chen, C. F., Baker, P. R., Chen, P. L., Kaiser, P., and Huang, L. (2007) Mass spectrometric characterization of the affinity-purified human 26S proteasome complex. Biochemistry 46, 3553–3565

B Andersen, K. M., Madsen, L., Prag, S., Johnsen, A. H., Semple, C. A., Hendil, K. B., & Hartmann-Petersen, R. (2009). Thioredoxin Txnl1/TRP32 Is a Redox-active Cofactor of the 26 S Proteasome. The Journal of Biological Chemistry, 284(22), 15246–15254. http://doi.org/10.1074/jbc.M900016200
