# Supplementary material for: The PSMA8 subunit of the spermatoproteasome is essential for proper meiotic exit and mouse fertility
Source: PLoS Genet. 2019 Aug 22;15(8):e1008316. doi: 10.1371/journal.pgen.1008316 (PMC6726247; doi:10.1371/journal.pgen.1008316)
Supplement: S6 Table — (PDF) [file pgen.1008316.s023.pdf]

S6 Table: Selection of some of the proteasome-related proteins co-immunoprecipitated with PSMA8 selected after analysis and filtering of the data.

Name	Uniprot Accession ID	No of unique peptides			Sequence coverage			iBAQ Intensity		
		Ab1	Ab2	Control	Ab1	Ab2	Control	Ab1	Ab2	Control
**E3 ligases**										
CAND1	Q6ZQ38	10	8	5	10	7.9	5.2	275300	143200	27601
CCT2	P80314	14	5	7	37.2	15	21.3	1713000	367550	184630
CUL3	Q9JLV5	3	6	1	6.8	9.6	1.3	104730	187950	6432.1
CUL9	E9QP09	3	2	0	1.1	0.6	0	20528	4934	0
NEDD4	P46935	2	0	0	4.7	0	0	68196	0	0
RAD18	E9Q392	3	0	0	43.7	0	0	20227000	0	0
RBX1	P62878	0	2	0	0	17.6	0	0	192900	0
SKP1	Q9WTX5	2	1	1	10.4	7.4	7.4	503960	136060	60179
TRIP12	A0A087WRV6	3	0	0	4.7	0	0	11882	0	0
TRIM36	E9Q3A0	5	1	0	9.3	2	0	237780	18278	0
UBR5	E9Q2H1	2	0	0	1.1	0	0	17518	0	0
UFL1	Q8CCJ3-1	2	0	0	3.8	0	0	57646	0	0
ZC3HC1	D3Z3D0	0	2	0	0	7.4	0	0	50351	0
**Deubiquitinases**										
USP5	Q3U4W8	1	2	0	1.9	4.1	0	3715.8	28815	0
USP7	F8VPX1	5	0	0	5.7	0	0	105410	0	0
USP9X	Q4FE56	10	2	0	4.9	1	0	121220	7683.3	0
USP14*	E9PYI8	0	1	0	0	5.2	0	0	32751	0
USP34	F6WJB7	6	0	0	2	0	0	41209	0	0
USP40*	Q8BWR4-3	1	1	0	1	1	0	22401	0	0
USP47	A0A1L1SV73	2	1	0	2.5	1.2	0	18169	2177.4	0
**Chaperones**										
AHSA1	Q8BK64	2	1	1	8.6	3	6.5	227160	2825.1	20386
CCT6B	Q61390	3	1	0	12.1	4.9	3.2	132900	25309	0
DNAJA1	P63037	2	1	0	8.1	3	0	1047200	67368	0
DNAJB9	Q9QYI6	1	2	0	4.5	11.3	0	66228	60676	0
DNAJC7	Q9QYI3	13	8	2	31.6	18.2	4	1376900	227200	25321
HSP90B1	P08113	17	8	9	28.8	12.2	14.5	4511300	1355800	619050
HSP90AB1	P11499	8	5	5	23.9	19.8	19.6	3171500	1127200	452050
HSPBP1	A0A0U1RPF2	2	0	0	6.9	0	0	46940	0	0
PSMG1 (PAC1)	Q9JK23	2	1	1	5.9	3.5	3.5	258480	35351	19395
TRAP1	Q9CQN1	4	5	0	6.9	8.1	0	143070	57267	0
**Putative PIPs/ a priori unrelated / spermatogenesis related**										
ADAD1	F8WI80	3	0	0	10	0	0	202110	0	0
BOLL	G3UYE8	3	1	1	11.7	3.2	3.2	400070	23474	37352
CAP1	P40124	0	2	2	0	4	6.8	0	46407	28930
CDK1	P11440	1	1	0	8.1	6.4	0	7093.3	41663	0

CDK5	P49615	3	1	2	12	6.5	6.5	542280	197000	29690
CDK16	Q04735-2	0	2	0	1.7	6.3	0	0	25675	0
DAZL	Q64368	3	3	0	13.4	15.4	0	473450	206150	0
MAEL	A0A0A6YWQ9	2	0	0	4.8	0	0	247850	0	0
RNF17	Q99MV7	8	4	1	5.7	2.9	0.7	161450	29164	4053.3
SHCBP1L	Q3TTP0	13	5	4	24.4	11	9.5	6787500	299050	173530
SMC4	E9Q2X6	4	1	0	3.6	0.7	0	130810	9056.2	0
SMC6	Q924W5	2	0	0	2.3	0	0	17639	0	0
SPAG1	Q80ZX8-3	4	3	0	8.1	6.1	0	290980	30285	0
SPATA5	A0A0G2JFY0	9	2	0	17.5	3.1	0	239890	13235	0
SPATA20	Q80YT5	7	7	3	11.6	12.4	4.1	1291900	234350	63556
SYCP1	Q62209	11	0	0	13.7	0	0	727550	0	0
TDRD1	Q99MV1	4	1	0	4.2	0.9	0	60474	3896.1	0
TDRD6	F2Z429	18	8	3	11.3	5	1.6	586920	46319	10974
TDRD9	Q14BI7	8	1	0	7.4	0.8	0	133480	5407.1	0
TDRKH	A0A0G2JFB2	4	2	0	9.4	4	0	384790	71610	0
TRIP13	Q3UA06	2	2	0	5.3	5.3	0	98523	41275	0

*Due to their relevance, these proteins were also included in the present table as an “ad-hoc” selection to show their behavior in spite of them not passing our cut-off.
