# Supplementary material for: The PSMA8 subunit of the spermatoproteasome is essential for proper meiotic exit and mouse fertility
Source: PLoS Genet. 2019 Aug 22;15(8):e1008316. doi: 10.1371/journal.pgen.1008316 (PMC6726247; doi:10.1371/journal.pgen.1008316)
Supplement: S7 Table — They have been classified in cells with small or large aggregates (n = 2 mice). (PDF) [file pgen.1008316.s024.pdf]

S7 Table. Quantification of the percentage of spermatocytes showing SYCP3 aggregates during prophase I stages in squash of seminiferous tubules of *Psma8*$^{+/+}$ and *Psma8*$^{-/-}$ testis. They have been classified in cells with small or large aggregates (n=2 mice).

		WT	KO	p-value
**Leptotene**	Small	75.3 ± 13.6	58.2 ± 9.4	0,28162
	Large	16.5 ± 10.5	17.8 ± 9.2	0,90562
**Zygotene**	Small	30.3 ± 26.3	22.5 ± 0.5	0,71665
	Large	9.1 ± 2.6	60.6 ± 5.2	0,00630 **
**Pachytene**	Small	8.8 ± 3.1	24.5 ± 3.9	0,04666
	Large	0.4 ± 0.1	11.3 ± 12.3	0,33571
**Diplotene**	Small	0.2 ± 0.3	1.3 ± 0.6	0,14999
	Large	0.0 ± 0.0	0.0 ± 0.0	-
