# Supplementary material for: The PSMA8 subunit of the spermatoproteasome is essential for proper meiotic exit and mouse fertility
Source: PLoS Genet. 2019 Aug 22;15(8):e1008316. doi: 10.1371/journal.pgen.1008316 (PMC6726247; doi:10.1371/journal.pgen.1008316)
Supplement: S2 Text — (DOCX) [file pgen.1008316.s026.docx]

**S2 Text. Supporting Information References**

1. Parra MT, Viera A, Gomez R, Page J, Carmena M, Earnshaw WC, et al. Dynamic relocalization of the chromosomal passenger complex proteins inner centromere protein (INCENP) and aurora-B kinase during male mouse meiosis. Journal of cell science. 2003;116 (Pt 6):961-74.

2. Gomez R, Jordan PW, Viera A, Alsheimer M, Fukuda T, Jessberger R, et al. Dynamic localization of SMC5/6 complex proteins during mammalian meiosis and mitosis suggests functions in distinct chromosome processes. Journal of cell science. 2013;126(Pt 18):4239-52.
